# Quantitative Trait Locus and Haplotype Analyses of Wild and Crop-Mimic Traits in U.S. Weedy Rice

**DOI:** 10.1534/g3.113.006395

**Published:** 2013-06-01

**Authors:** Muhamad S. Mispan, Lihua Zhang, Jiuhuan Feng, Xing-You Gu

**Affiliations:** Plant Science Department, South Dakota State University, Brookings, South Dakota 57007

**Keywords:** weedy rice, adaptation, crop mimicry, quantitative trait locus, haplotype

## Abstract

Conspecific weeds retained characteristics from wild ancestors and also developed crop mimicries for adaptation and competitiveness. This research was conducted to identify quantitative trait loci (QTL) associated with the wild and crop-mimic traits and to determine haplotype variants for QTL-rich regions in U.S. weedy rice. An F_2_ population from the cross between a cultivated (EM93-1) and a U.S. weedy (US1) rice line was evaluated for six wild and eight crop-mimic traits in a greenhouse to identify the QTL. A core collection of 27 U.S. weedy red rice lines and 14 AA-genome wild rice lines were determined for the haplotype variants. A total of 49 QTL were identified, with 45 collocated as clusters on 14 genomic segments. The number of haplotypes across the 14 segments was lower in the weedy (6.1 ± 2.4) than in the wild (7.5 ± 1.8) rice sample. Both samples shared ~50% haplotypes (wild-like). The EM93-1−like haplotypes accounted for a greater proportion (30 ± 26%) of the haplotypes in the weedy than in the wild (7 ± 10%) rice. Based on haplotype patterns for the 14 QTL cluster regions, 26 of the 28 red rice lines were clustered into two groups corresponding to the black-hull awned and straw-hull awnless morphological types, respectively. The QTL analysis demonstrated that conspecific weed-crop differentiation involved many genomic segments with multiple loci regulating natural variation for adaptation and competitiveness. The haplotype analysis revealed that U.S. weedy rice retained large blocks of linkage disequilibrium for the multiple loci from the wild relatives and also incorporated haplotypes from cultivars.

Weeds are unwanted plants that have adapted to human-disturbed environments and compete with crops for limited natural resources (Harlan 1965; Baker 1974; Booth *et al.* 2003). Many crop species, such as barley (*Hordeum vulgare*), oat (*Avena sativa*), rice (*Oryza sativa*), sorghum (*Sorghum bicolor*), and wheat (*Triticum aestivum*), have conspecific or congeneric weeds (Ellstrand *et al.* 1999). Crops and their conspecific weeds share evolutionary origins and produce fertile hybrids resulting in gene flow from cultivars to the local weed populations. Conspecific weeds retained characteristics from wild relatives (*e.g.*, seed shattering and dormancy) and also developed crop mimicries (*e.g.*, plant/seedling morphologies and rapid vegetative growth) to enhance the adaptation and competitiveness in agro-ecosystems. Knowledge of specific genes regulating natural variation for the wild and crop mimic traits is essential to understand weed evolution and adaptation and to develop weed management and transgene mitigation strategies.

Weedy rice refers to various forms of unwanted plants that belong to the *Oryza* genus and are phenotypically intermediate between cultivated and wild (*Oryza* spp.) rice (Oka 1988; Delouche *et al.* 2007). Asian cultivated rice (*O. sativa*) originated from the wild ancestors (*O. rufipogon* and *O. nivara*) at multiple sites in South to Southeast Asia and differentiated into the *indica* and *japonica* subspecies, which are cultivated mainly in tropical and temperate areas, respectively (Kush and Brar 2002). Based on diagnostic characteristics for the subspecific and the wild-cultivated variation, weedy rice could be classified into *indica*- and *japonica*-like groups, with each consisting of wild-like and crop-mimic subgroups (Tang and Morishima 1997; Suh *et al.* 1997). Red rice (*i.e*., red or brown pericarp-colored weedy rice) prevails in both *indica*- and *japonica*-like weed populations. The *indica*-like weed populations in Tropical Asia, where there was/is wild rice, could originate from natural hybridization between cultivated and wild rice or from natural variants of wild rice (Oka 1988). The *japonica*-like and some *indica*-like weed populations in the areas historically absent of wild rice (*e.g.*, Europe and the United States) may originate from old cultivars adaptive to the local environmental conditions or natural hybridization between *indica* and *japonica* cultivars (Tang and Morishima 1997; Suh *et al.* 1997; Ishikawa *et al.* 2005; Cao *et al.* 2006; Londo and Schaal 2007). U.S. weedy rice was likely derived from contaminants in imported seeds of commercial rice (Delouche *et al.* 2007) and has population structures closest to the *indica* and *aus* ecotypes of the *indica* subspecies (Reagon *et al.* 2010).

Quantitative trait locus (QTL) analysis, map-based cloning, and gene-based haplotype analysis have been used to identify genes and their genomic organization or haplotype patterns for wild and crop mimic traits differentiated between weedy and cultivated rice. A total of 26 QTL were identified for 16 traits from a cross between a *japonica* cultivar and a *japonica*-like weedy rice line from France (Bres-Patry *et al.* 2001) and 20 QTL identified for five wild traits from a cross between an *indica* cultivar and a Thailand *indica*-like weedy rice line (Gu *et al.* 2005a; Ye *et al.* 2010). Many of the reported QTL were collocated on a small number of chromosomal segments. Some collocated QTL (weed adaptive haplotypes), such as *qSD7-1/qPC7* or *qSD4/qHC4* for seed dormancy and pericarp or hull color, could not be broken across several generations (Gu *et al.* 2005b). The *qSD7-1/qPC7* QTL were map-based cloned as a single gene (Gu *et al.* 2011), which is the red pericarp gene *Rc* encoding a bHLH family transcription factor (Sweeney *et al.* 2006; Furukawa *et al.* 2007). The functional *Rc* alleles in weedy red rice were clustered into two groups corresponding to the *indica* and *japonica* subspecies and the *Rc* alleles in U.S. red rice originated more likely from *indica* landraces than from wild rice (Reagon *et al.* 2010; Gu *et al.* 2011). Haplotype analysis for the seed shattering genes *qsh1* (Konishi *et al.* 2006) and *sh4* (Li *et al.* 2006) suggested that these loci did not contribute to the trait variation in U.S. weedy rice populations (Thurber *et al.* 2010). Similarly, the haplotype diversity for the *semi-dwarf1* (*sd1*) region was not associated with the phenotypic variation for plant growth-related traits (Reagon *et al.* 2011).

Despite the relatively short history of rice cultivation in the United States, weedy rice populations in the country have differentiated into many ecotypes and structures. Collected ecotypes are often classified based on seed morphologies (*e.g.*, pericarp and hull colors, awn presence/absence and grain types) and vary for multiple wild and crop mimic traits (Noldin *et al.* 1999; Delouche *et al.* 2007). The population structures were defined by genomic patterns for randomly selected short-DNA sequences (Reagon *et al.* 2010). There is still a considerable lack of knowledge on genes and genome structures underlying the trait variation and ecotype differentiation in weedy rice. Distinguishing from neutrally evolving DNA sequences, QTL for adaptive traits are targets of natural selections. Therefore, the objectives of this research were to identify QTL for wild and crop mimic traits, including those enhancing the competitiveness during the vegetative growth phase, and to determine haplotype patterns for the QTL-containing regions in U.S. weedy rice.

## Materials and Methods

### Plant materials

An F_2_ population from the ‘EM93-1’/‘US1’ cross was developed for QTL mapping. EM93-1 is a semi-dwarf line of *indica*-type cultivated rice and carries the mutant allele at *sd1* (Ye *et al.* 2013). US1 is a pure line of *indica*-like weedy rice from the United States and was used in the reported research (Tang and Morishima 1997, Suh *et al.* 1997). A core collection of 27 U.S. weedy rice lines and 14 lines of the AA-genome wild rice (*O. rufipogon*, *O. nivara*, and *O. glumipatula*), which were introduced from the National Small Grains Collection, the United States Department of Agriculture–Agriculture Research Service, were used to identify haplotype variants for mapped QTL regions. All the 28 (include the parent US1) weedy rice lines are red rice and represent four morphological types: black hull awned (BHA), furrow hull awned (FHA), straw hull awned (SHA), and straw hull awnless (SH). These weedy rice lines and some wild rice lines were planted in a greenhouse to confirm the seed morphologies (see Supporting Information, Table S1, Figure S1).

### Plant cultivation

Fully after-ripened (dormancy-released) seeds from the F_1_ and parental plants were germinated in a 30° incubator for 5 d and newly germinated seeds planted into 200-cell Plug trays filled with the rice nutrient solution (Yoshida *et al.* 1976) for 4 weeks to synchronize seedling size. About 500 F_2_ seedlings were transplanted into pots (12 × 12 × 15 cm dimensions), with one plant per pot. The pots were filled with clay soil mixed with greenhouse medium (Sunshine Growth Mix) and placed in water-tight containers (60 cm ×120 cm) in a greenhouse. The greenhouse temperatures were set at 30/23° for day/night and the day length was natural, except for the period from 35 to 70 d after germination when a short-day (10 hr) treatment was applied to induce floral initiation. Flowering time was recorded as the date when the first panicle in a plant emerged from the leaf sheath. Seeds were harvested at 40 d after flowering, air-dried in the greenhouse for 3 d before stored in a freezer (-20°) to maintain the dormancy status.

### Phenotypic identification for wild and crop-mimic traits

The 14 traits were measured with one or more parameters as described in Table 1. Methods used to measure the wild traits—seed shattering (SH), seed dormancy (SD), awn (AN), and hull color (HC)—were similar to those previously described (Gu *et al.* 2005a). Three reproductive tillers cut from a plant were gently shaken in a bucket for 10 sec to collect shattered seeds, and shattered and nonshattered seeds were counted to estimates a shattering rate for the plant. A sample of ~30 seeds in a 9-cm Petri dish lined with a filter paper and wetted with 5 mL of water was germinated at 30° and 100% relative humidity in dark for 7 d. The mean germination (%) of three samples was used to estimate the degree of seed dormancy for a plant. AN was quantified by the mean awn length and percentage of seeds with awn for a sample of ~50 seeds from a plant. HC was measured with the ChromaMeter Minolta CR310, which transfers reflectance spectra into the L*, a*, and b* readings to quantify blackness, redness, and yellowness, respectively. L* ranges from 0 to 100, with 0 and 100 indicating completely nonreflective (black) and perfectly reflective (white), respectively. a* varies from −100 to 100, with negative and positive values indicating degrees of green and red pigmentations, respectively; a high, positive a* value indicates a high intensity of redness. b* varies from −100 to 100, with the negative and positive values indicating degrees of blue and yellow pigmentations, respectively; a high, positive b* value indicates a high intensity of yellow pigmentation. The pigment measurement was conducted using >100 seeds in a 6-cm Petri dish on a dark background and repeated three times.

**Table 1:** List of wild and crop-mimic traits evaluated for the F_2_ EM93-1/US1 population

Traits (abbr.)	Description	Measurement
Wild-like		
Leaf sheath color (LSC)	Pigments on the leaf sheath	Visualized as purple or green
Hull color (HC)	Pigments on the hull (lemma and palea)	Visualized as black and straw and also quantified with reflectance spectra (L*, a*, and b*)
Pericarp color (PC)	Pigments on the pericarp tissue	Visualized as red, brown, or white
Awn (AN)	Long stick appendage with the lemma	Average AN length and percent seeds with AN
Shattering (SH)	Seeds shattered during maturation	Percentage seeds shattered
Seed dormancy (SD)	Delayed germination	Germination % of air-dried seeds
Crop mimicry		
Plant height-increasing rate (PHR)	Rate of increase in plant height	Differences in plant height between periods of 4, 6, and 8 wk after germination
Tiller number-increasing rate (TNR)	Rate of increase in number of tillers	Differences in no. of tillers between periods of 4, 6, and 8 wk after germination
Flowering time (FT)	Time period required for a plant to flower	Days to the emergence of the 1st panicle from the leaf sheath
Plant height (PH)	Height of a matured plant	Length of the main stem from the base to the top of the panicle
Reproductive tiller (RTN)	Tillers with seeded panicles	No. of reproductive tillers in a plant
Seed number (SN)	Fertile florets in a plant.	The total number of seeds
Seed setting percentage (SSP)	Percentage of filled seeds for a plant	Percentage of fertile/total florets averaged over three main panicles
Seed weight (SW)	Dry weight of seeds from a plant	Weight (g) of 100 air-dried seeds

The crop-mimic traits include plant height (PHR)- or tiller number (TNR)-increasing rates and seed setting percentage (SSP). Plant height and the number of tillers per plant were observed at 4, 6, and 8 wk after germination, and PHR and TNR were estimated as ratios of the observations between week 6 and week 4 or week 8 and week 6. SSP was evaluated as the proportion of the number of fertilized seeds to the total number of spikelets on the three panicles used to measure the shattering rate.

### Marker genotyping and linkage map construction

Genomic DNA was extracted from fresh leaf segments of the parental and F_2_ seedlings. Approximately 320 rice microsatellite markers (McCouch *et al.* 2002) were screened for polymorphism between the parents. Polymorphic markers were used to genotype a subpopulation of 188 F_2_ plants to develop a framework linkage map to scan for QTL. The remaining F_2_s were genotyped only with the markers nearest to QTL peak positions to confirm their effects. DNA extraction, marker amplification by polymerase chain reaction, and marker display by electrophoresis with nondenatured polyacrylamide gel were performed using the previously described methods (Gu *et al.* 2004a). The linkage map was constructed using MAPMAKER/EXP 3.0 (Lincoln *et al.* 1992). The marker genotyping data were also analyzed for segregation distortion by a chi-square test.

### QTL mapping

The initial QTL analysis was conducted based on the subpopulation. The composite interval mapping program of the WinQTLCart software (Wang *et al.* 2006) was used to generate likelihood ratio distributions for individual traits to infer QTL positions and to estimate QTL additive (*a*) and dominance (*d*) effects. The QTL threshold was established by 1000 random permutations at a genome-wide type I error of 5%. The degree of dominance for a QTL was estimated by the *d/a* ratio. A QTL confidence interval was expressed by the length of 1-LOD (or 4.61 likelihood ratio) support region in centimorgan derived from Kosambi’s map function. Two or more QTL with overlapping 1-LOD support intervals were defined as a QTL cluster.

The QTL detected by composite interval mapping in the subpopulation were corroborated by single marker analysis (SMA) for data from the full population of ~480 F_2_ plants. The SMA was conducted using one-way analysis of variance with the marker nearest to a QTL peak as the indicator variable. The variables were coded as 0, 1, and 2 for the EM93-1-like homozygote, heterozygote, and US1-like homozygote, respectively, for the analysis. Analysis of variance was performed using the SAS GLM procedure (SAS Institute 2011).

### Haplotype analysis

Haplotype analysis was conducted for the QTL cluster regions detected from the mapping population. The analysis was limited to the weedy and wild rice lines (Table S1) and did not include cultivars because some wild characteristics in weeds, such as a high degree of seed shattering and dormancy, black hull color, and long awn (Figure S1), are absent or very rare in cultivars. The 27 weedy and 14 wild rice lines were genotyped with all markers mapped on each of the genomic regions that encompass 1-LOD support intervals of the clustered QTL. The marker genotypes (nonallelic combinations) were used to determine haplotype variants for individual QTL cluster regions. The physical length of a haplotype was estimated based on marker positions on the Nipponbare genome sequence (Gramene 2012). The weedy and wild rice samples were compared for the number of haplotypes using a paired Student’s *t*-test. The marker genotyping data were subjected to a cluster analysis using the single linkage method (SAS Institute 2011) to group the U.S. weedy red rice lines and to infer phylogenetic relations among the weedy and wild rice lines.

## Results

### Phenotypic variation and correlation

**Figure 1:**
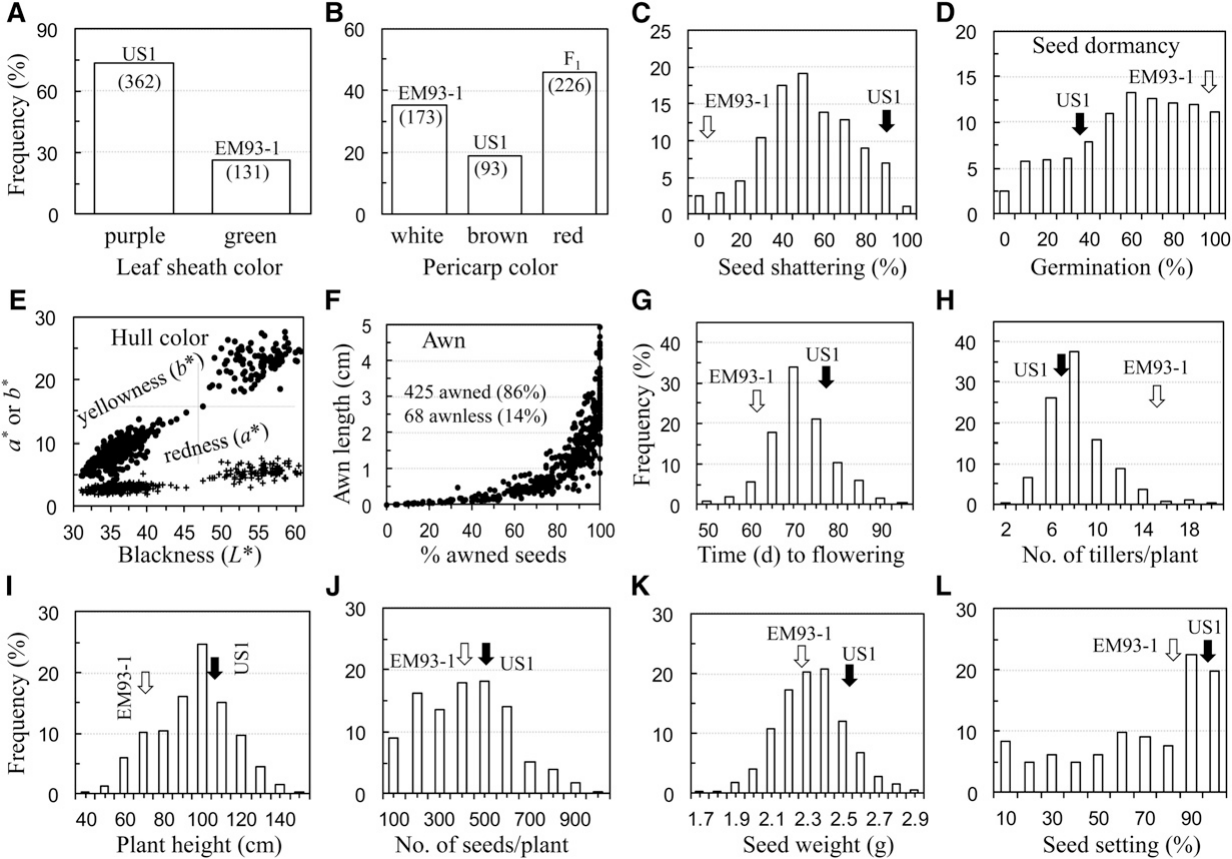
Frequency distributions of wild (A−F) and crop-mimic (G−L) traits in the F_2_ EM93-1/US1 population. Arrows indicate the parental means.

The parental lines US1 and EM93-1 were divergent in phenotype for the six wild traits, which varied in segregation pattern in the F_2_ population. Both leaf sheath (LSC) and pericarp (PC) colors were scored as qualitative traits; the F_2_ plants could be grouped into the two (purple and green) types for LSC or three (red, brown, and white) types for PC (Figure 1, A and B). The purple or brown type was US1-like, whereas the green or white type was EM93-1-like for LSC or PC. The red type of the F_2_s resembled the hybrid F_1_s for PC, presumably resulting from the complementation of non-allelic genes assembled from the two parents. Both SH and SD are quantitative traits and their F_2_ frequency distributions approximated to normal distributions (Figure 1, C and D). The HC and AN traits appeared to be of both qualitative and quantitative natures. For HC, the F_2_ population consisted of black, brown, furrow, golden, and straw hull−colored plants and some plants had the hull tissue with multiple colors. However, using the component reflectance spectrum for blackness (L*) or yellowness (b*), the F_2_s could be divided into basically two groups, although variation occurred within each group (Figure 1E). For AN, the F_2_ population consisted of ~86% awned and ~14% awnless plants. However, the awned plants varied in awn length from ~0.5 to 5 cm or percentage of awned seeds from ~2 to 100%, and there was a nonlinear correlation between these two quantitative measurements (Figure 1F).

The parent US1 had later flowering time (FT), less reproductive tillers/plant (RTN), and taller plant height (PH) than the parent EM93-1 and was similar to EM93-1 in seed numbers/plant (SN), seed weight (SW), and seed setting percentage (SSP). In the F_2_ population, FT, PH, RTN, SN, and SW displayed approximately normal frequency distributions and transgressive segregation (Figure 1, G−K), and only SSP had a negatively skewed frequency distribution (Figure 1L).

**Figure 2:**
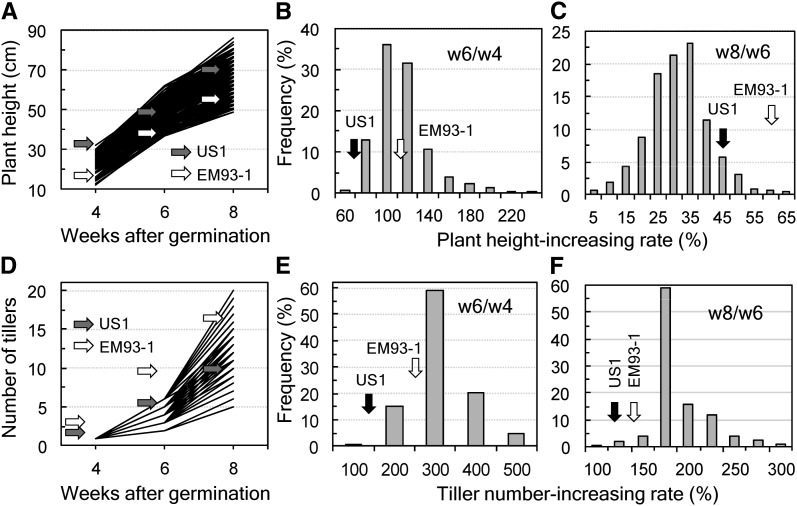
Dynamic patterns of plant vegetative growth in the F_2_ EM93-1/US1 population. (A, D) Plant height and the number of tillers per plant at weeks 4, 6, and 8. (B−C, E−F) Frequency distributions of the plant height- and tiller number-increasing rates for week 6/week 4 (w6/w4) or w8/w6. Arrows indicate the parental means.

Phenotypic variation for the plant vegetative growth-related traits (*i.e.*, plant height and tiller number) was observed between the two parental lines and among the F_2_ individuals. The range of the observed variations increased with times from week 4 to week 8 in the F_2_ population (Figure 2, A and D). Both PHR and TNR exhibited approximately normal frequency distributions for each of the two (weeks 4−6 and weeks 6−8) periods (Figure 2, B and C, E and F).

Each of the 14 traits measured was correlated with three (HC) to 10 (AN) of the remaining 13 traits. The correlation occurred between wild traits, crop-mimic traits, and wild and crop-mimic traits (Table 2). For example, SD was correlated with the other five wild traits and also with the two crop-mimic traits FT and SW; and the two vegetative growth-related traits PHR and TNR were negatively correlated (*r* = -0.21). The phenotypic correlations suggest that none of the adaptive traits was inherited independently.

**Table 2:** Summary of correlation coefficients (*r*) between adaptive traits segregating in the F_2_ EM93-1/US1 population

		Wild Trait*^b^*						Crop-Mimic Trait							
Trait*a*		SH	SD	PC	HC	AN	LSC	PH	FT	RTN	SN	SW	SSP	PHR	TNR
Wild-like	SH		**<0.002**	**0.035**	0.195	**<0.001**	0.914	**0.041**	**<0.011**	**<0.009**	**<0.001**	**0.035**	**<0.001**	0.178	0.731
	SD	**0.14**		**<0.001**	**<0.001**	**<0.001**	**<0.003**	0.447	**0.023**	0.329	0.114	**0.035**	0.176	0.141	0.997
	PC	**0.10**	**0.19**		0.209	**<0.008**	0.190	**<0.001**	**<0.001**	**<0.001**	**<0.007**	0.481	**0.022**	0.676	0.606
	HC	−0.06	**-0.20**	−0.04		**<0.001**	**0.023**	0.073	0.298	0.547	0.063	0.749	0.954	0.844	0.762
	AN	**0.18**	**0.22**	**0.12**	**-0.23**		0.222	**<0.001**	**<0.001**	**<0.005**	**<0.001**	**<0.001**	**<0.001**	0.936	0.798
	LSC	0.00	**0.13**	0.06	**-0.14**	−0.06		0.082	**<0.001**	0.963	0.103	**0.035**	0.126	**<0.001**	**<0.001**
Crop mimic	PH	**0.09**	−0.03	**0.19**	−0.08	**0.23**	0.08		0.451	**<0.001**	**<0.001**	0.055	**<0.001**	**<0.001**	**<0.005**
	FT	**0.12**	**-0.10**	**0.43**	−0.05	**0.20**	**-0.37**	−0.03		**<0.001**	0.942	**<0.001**	0.971	**<0.001**	0.220
	RTN	**-0.12**	0.04	**-0.20**	0.03	**-0.13**	0.00	**-0.42**	**-0.22**		0.609	**<0.001**	0.249	**<0.001**	**<0.001**
	SN	**0.21**	0.07	**0.12**	−0.01	**0.28**	0.07	**0.28**	0.00	−0.02		**0.019**	**<0.001**	**0.047**	0.731
	SW	**0.10**	**0.10**	0.03	0.08	**0.40**	**-0.10**	0.08	**0.16**	**-0.17**	**0.12**		**<0.001**	0.657	0.541
	SSP	**0.19**	0.06	**0.10**	−0.06	**0.27**	0.07	**0.15**	0.00	−0.05	**0.56**	**0.19**		0.153	0.366
	PHR	0.06	0.07	0.02	−0.01	0.00	**0.23**	**0.26**	**-0.20**	**-0.15**	**0.09**	0.02	0.06		**<0.001**
	TNR	0.02	0.00	−0.02	0.01	−0.01	**-0.13**	**-0.30**	0.06	**0.26**	−0.02	−0.03	−0.04	**-0.21**	
Pair*^c^*		9	7	8	3	10	6	8	8	8	7	8	6	6	4

SH, seed shattering (shattering rate); SD, seed dormancy (% germination); PC, pericarp color (3-color measurement); HC, hull color (L*); AN, awn (awn length); LSC, leaf sheath color; PH, plant height; FT, flowering time; RTN, number of reproductive tillers/plant; SN, no. of seed/plant; SW, seed weight; SSP, seed setting percentage; PHR, the week6/week4 plant height ratio; and TNR, the week6/week4 tiller number ratio.

a Traits were evaluated for 480-493 F_2_ plants.

b Listed below and above the diagonal line are *r* values and their probability (*P*) levels, respectively. Values significant at *P* < 0.05 are shown in bold.

c Number of trait pairs with a significant correlation in the column.

### The framework linkage map

**Figure 3:**
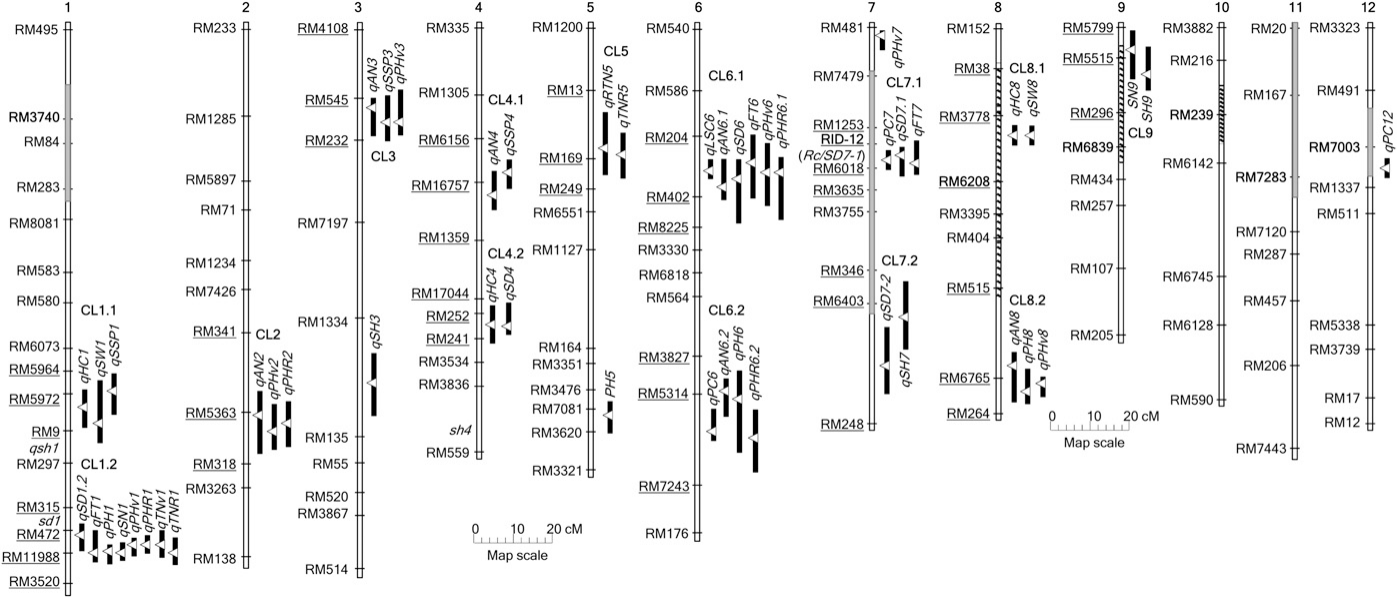
Framework linkage map and QTL distribution. The map was constructed based on 188 plants from the F_2_ population. Vertical bars represent 12 chromosomes marked with rice microsatellite loci. Map distance in centiMorgan was derived from Kosambi’s map function. Chromosomal segments with markers displaying a segregation distortion are depicted by gray-colored or striped bars, which indicate the distortion in favor of the alleles from the parental line EM93-1 or US1. The marker in bold indicates its segregation ratio deviated most from the Mendelian expectation (Table S2). The lengths of black bars right to each chromosome indicate 1-LOD support regions for the named QTL associated with wild and crop mimic traits. The empty triangles point to the peak position of the QTL in the chromosome. QTL with overlapping 1-LOD support regions are grouped as a QTL cluster (CL). Underlined markers were used to determine haplotype patterns for the QTL cluster regions in weedy and wild rice lines (Table S1). Known genes *qsh1*, *sd1*, *sh4*, *Rc*, and *SD7-1* were positioned on the map according to their physical positions on the rice reference genome sequence (Gramene 2012).

Of the 320 markers screened, 173 are polymorphic between EM93-1 and US1, with the polymorphic rate being 54%. The subpopulation of 188 F_2_ plants was genotyped with 123 polymorphic markers and the genotyping data (no missing data) were used to construct a framework linkage map (Figure 3). The total length of the map was 1423 cM, with the average inter-marker distance being 13 (±7) cM. Segregation distortion was detected for markers on seven segments of chromosomes 1 and 7 to 12. Four and three of the seven segments had allelic frequencies in favor of EM93-1- and US1-derived alleles, respectively (Figure 3, see Table S2).

### QTL associated with wild and crop-mimic traits

A total of 21 QTL were associated with the six wild traits in the subpopulation, including one for LSC, three for PC, three for HC, six for AN, three for SH, and five for SD (Table 3, see Figure S2, A−F). The number of QTL for a trait varied with the measurements. For example, only one QTL for PC (*qPC7*, *R^2^* = 0.59) or HC (*qHC4*, *R^2^* = 0.69) was detected when plants were scored as the presence (1) or absence (0) of the pigments. *qPC7* was confirmed and additional two loci (*R^2^* < 0.09) were detected for PC when it was scored as red (2), brown (1), and white (0). Similarly, *qHC4* was confirmed and additional two HC loci (*R^2^* <0.09) were detected when the trait was quantified with the reflectance spectra (L*, a*, and b* values). Likewise, three awn loci (*i.e.*, *qAN4*, *6.1*, and *8*) were associated with both awn length and percentage of awned seeds and the remaining three QTL (*i.e.*, *qAN2*, *3*, and *6.2*) associated with only one of the two measurements. The parent EM93-1 contributed alleles increasing seed dormancy for *qSD1.2*, black pigment for *qHC8*, red pigment for *qPC6*, and awn length or awned-seed for *qAN6.1*, whereas the parent US1 contributed the effect-increasing alleles to the remaining 17 QTL. Approximately one half of the 21 QTL had a strong dominance effect (*d*) on PC (*qPC6* and *7*), HC (*qHC1*, *4* and *8*), AN (*qAN6.2*), SH (*qSH7*), or SD (*qSD4* and *7.2*), as suggested by the *d*/*a* ratios of ≥1 or ≤ -1 (Table 3).

**Table 3:** List of QTL associated with wild traits in the F_2_ EM93-1/US1 population

		CIM (N = 188)*^b^*						SMA (N > 480)*^c^*		
QTL*^a^*	Ch.	Peak	LR	*a*	*d*	*d/a*	*R^2^*	Marker	*P*	*R^2^*
Leaf sheath color										
* qLSC6*	6	35	102	0.8	0.5	0.6	0.42	RM204	<0.0001	0.31
Pericarp color										
* qPC6* (red/brown/white)	6	122	13	−0.1	0.3	−3.0	0.03	RM5314	0.0001	0.01
* qPC7* (present/absent)	7	39	236	0.2	0.5	2.5	0.59	RM6018	<0.0001	0.76
(red/brown/white)		35	368	0.9	0.9	1.0	0.84	RID12/*Rc*	<0.0001	0.83
* qPC12* (red/brown/white)	12	39	17	0.3	−0.1	−0.3	0.04	RM7003	0.0001	0.02
Hull color										
* qHC1* (blackness L*)	1	101	19	−0.74	−7.4	10.0	0.08	RM5972	0.0006	0.03
* qHC4* (visual score)	4	80	224	0.9	0.7	0.8	0.69	RM252	<0.0001	0.59
(blackness L*)		79	38	−9.1	−10.9	1.2	0.31	RM252	<0.0001	0.52
(redness a*)		79	75	−1.2	−1.4	1.2	0.39	RM252	<0.0001	0.48
(yellowness b*)		79	190	−7.4	−7.9	1.1	0.57	RM252	<0.0001	0.51
* qHC8* (blackness-L*)	8	33	50	21.5	20.8	1.0	0.01	RM3778	0.0038	0.02
Awn										
* qAN2* (% awned seeds)	2	108	12	9.2	5.5	0.6	0.02	RM5363	0.0101	0.02
* qAN3* (awn length)	3	25	22	0.7	−0.4	−0.6	0.11	RM545	0.0001	0.04
* qAN4* (awn length)	4	50	57	1.0	0.1	0.1	0.24	RM16757	<0.0001	0.11
(% awned seeds)		51	77	31.4	16.9	0.5	0.17	RM16757	<0.0001	0.09
* qAN6.1* (awn length)	6	40	24	−0.6	0.2	−0.3	0.12	RM204	0.0011	0.03
(% awned seeds)		40	19	−14.2	11.3	−0.8	0.12	RM204	0.0003	0.04
* qAN6.2* (awn length)	6	98	25	0.5	−0.5	−1.0	0.14	RM5314	<0.0001	0.06
* qAN8* (% awned seeds)	8	82	39	26.9	11.5	0.4	0.10	RM6765	<0.0001	0.11
(awn length)		81	28	0.7	−0.1	−0.1	0.11	RM6765	<0.0001	0.10
Seed shattering (%)										
* qSH3*	3	97	33	14.6	10.7	0.7	0.09	RM1334	<0.0001	0.08
* qSH7*	7	72	15	1.0	11.9	11.9	0.01	RM6403	0.0118	0.02
* qSH9*	9	12	12	8.5	−2.0	−0.2	0.07	RM5515	0.0102	0.02
Seed dormancy (% germination)										
* qSD1.2*	1	136	12	8.4	2.5	0.3	0.03	RM472	0.0099	0.02
* qSD4*	4	81	26	−11.0	11.2	−1.0	0.17	RM252	0.0051	0.02
* qSD6*	6	38	18	−11.0	7.7	−0.7	0.13	RM204	0.0302	0.01
* qSD7.1*	7	34	12	−5.2	−3.9	0.8	0.02	RID12	0.0004	0.03
* qSD7.2*	7	86	17	−4.5	24.1	−5.4	0.12	RM6403	0.0096	0.02

QTL, quantitative trait loci; Ch, chromosome; CIM, composite interval mapping; SMA, single marker analysis; ANOVA, analysis of variance.

a Measurements in parenthesis are used to detect the QTL.

b Likelihood ratio (LR) at the peak (cM) position, additive (*a*) and dominance (*d*) effects, and proportion of the phenotypic variance explained by the locus (*R*^2^) were computed by the CIM program based on a subpopulation of 188 (N) F_2_ plants.

c The marker nearest the peak was used to confirm the QTL with the whole population of 480-493 (N) F_2_ plants by SMA. The F-test probability level (*P*) and *R*^2^ were estimated by one-way ANOVA.

A total of 15 QTL were associated with the six crop-mimic traits, including three for FT, one for RTN, four for PH, two for SN, two for SW, and three for SSP, in the subpopulation (Table 4, see Figure S2, G−K). The QTL *qPH1* (*R^2^* = 0.38) and *qFT7* (*R^2^* = 0.13) had a relatively large effect on PH or FT, and the remaining 13 loci contributed <9% to the total phenotypic variances. The US1- and EM93-1−derived alleles increased additive effects for 12 and three of the 15 QTL, respectively. Of the 15 QTL, six displayed a strong dominance effect on FT, RTN, SW, or SSP (Table 4).

**Table 4:** List of QTLs associated with crop-mimic traits expressed during the reproductive growth phase in the F_2_ EM93-1/US1 population

		CIM (N = 188)*^b^* CIM						SMA (N > 480)*^c^*		
QTL*^a^*	Ch.	Peak	LR	*a*	*d*	*d/a*	*R^2^*	Marker	*P*	*R^2^*
Flowering time, d										
* qFT1*	1	143	12	−2.1	−1.0	0.5	0.02	RM11988	0.0049	0.02
* qFT6*	6	29	21	−2.8	−2.7	1.0	0.02	RM204	<0.0001	0.06
* qFT7*	7	41	99	5.8	3.3	0.6	0.13	RM6018	<0.0001	0.08
Number of reproductive tillers per plant										
* qRTN5*	5	31	17	0.2	−1.7	−8.5	0.07	RM169	<0.0001	0.06
Plant height, cm										
* qPH1*	1	142	255	22.3	9.2	0.4	0.38	RM472	<0.0001	0.17
* qPH5*	5	105	26	6.0	−0.1	−0.02	0.05	RM7081	<0.0001	0.04
* qPH6*	6	100	20	5.4	0.1	0.02	0.03	RM3827	0.0044	0.03
* qPH8*	8	95	20	5.1	−2.6	−0.5	0.05	RM6765	0.0001	0.04
Number of seeds per plant										
* qSN1*	1	143	31	98.3	81.5	0.8	0.02	RM11988	<0.0001	0.05
* qSN9*	9	5	13	65.1	3.0	0.05	0.05	RM5515	0.0091	0.02
100-seed weight, g										
* qSW1*	1	101	22	−0.1	−0.4	4.0	0.01	RM5972	0.0257	0.01
* qSW8*	8	33	248	1.2	1.2	1.0	0.03	RM3778	0.0038	0.02
Seed setting percentage										
* qSSP1*	1	87	12	0.1	−0.2	−2.0	0.07	RM6073	0.0274	0.01
* qSSP3*	3	30	12	0.2	−0.1	−0.5	0.06	RM545	0.0028	0.02
* qSSP4*	4	43	12	0.2	−0.2	−1.0	0.08	RM16757	<0.0001	0.05

QTL, quantitative trait loci; Ch, chromosome; CIM, composite interval mapping; SMA, single marker analysis; ANOVA, analysis of variance.

a Measurements in parenthesis are used to detect the QTL.

b Likelihood ratio (LR) at the peak (cM) position, additive (*a*) and dominance (*d*) effects, and proportion of the phenotypic variance explained by the locus (*R*^2^) were computed by the CIM program based on a subpopulation of 188 (N) F_2_ plants.

c The marker nearest the peak was used to confirm the QTL with the whole population of 480-493 (N) F_2_ plants by SMA. The F-test probability level (*P*) and *R*^2^ were estimated by one-way ANOVA.

Six QTL (*qPHv*) were associated with plant height at weeks 4, 6, or 8 of the vegetative growth phase (Table 5, see Figure S2, L and M). The *qPHv1* and *qPHv8* QTL were mapped to the same positions as *qPH1* and *qPH8*, respectively (refer to peak positions and nearest markers in Table 4 and Table 5), suggesting that *qPHv1* and *qPH1* and *qPHv8* and *qPH8* are underlain by same genes expressed in both of the vegetative and reproductive growth phases. The remaining four *qPHv* QTL appeared to express only in given period(s) of the vegetative growth phase. Four QTL were associated with plant height-increasing rate (PHR) and three (*qPHR1*, *2*, and *6.1*) of the four collocated with the *qPHv* loci (*qPHv1*, *2*, and *6*). The other PHR QTL (*qPHR6.2*) appeared to express only from weeks 4 to 6. US1 and EM93-1 contributed the effect-increasing alleles to the *qPHR1* and *6.1* and the *qPHR2* and *6.2* loci, respectively.

**Table 5:** List of QTL associated with crop-mimic traits expressed during the vegetative growth phase in the F_2_ EM93-1/US1 population

		CIM (N = 188)*^b^* CIM						SMA (N > 480)*^c^*		
QTL*^a^*	Ch.	Peak	LR	*a*	*d*	*d/a*	*R^2^*	Marker	*P*	*R^2^*
Tiller number										
* qTNv1* (w6)	1	139	56	−0.5	−0.2	0.4	0.14	RM472	<0.0001	0.04
(w8)		139	53	−1.7	−0.7	0.4	0.14	RM472	<0.0001	0.04
Tiller number-increasing rate, %										
* qTNR1* (w6/w4)	1	139	47	−52	−22	0.4	0.14	RM472	<0.0001	0.04
* qTNR5* (w8/w6)	5	33	12	10	4.7	0.5	0.04	RM169	0.0071	0.01
Plant height, cm										
* qPHv1* (w4)	1	142	99	3.0	−0.5	−0.2	0.43	RM472	<0.0001	0.12
(w6)		142	106	6.5	−0.5	−0.1	0.38	RM472	<0.0001	0.14
(w8)		140	78	8.5	−0.1	−0.01	0.29	RM472	<0.0001	0.09
* qPHv2* (w6)	2	118	40	−3.6	0.2	−0.1	0.11	RM5363	<0.0001	0.08
(w8)		114	32	−5.1	0.3	−0.1	0.11	RM5363	<0.0001	0.06
* qPHv3* (w4)	3	30	16	0.5	−1.1	−2.2	0.06	RM545	<0.0001	0.06
* qPHv6* (w6)	6	37	21	2.8	0.3	0.1	0.06	RM204	<0.0001	0.04
(w8)		36	21	4.4	1.0	0.2	0.06	RM204	0.0004	0.03
* qPHv7* (w8)	7	1	12	−1.7	1.4	−0.8	0.05	RM481	0.0012	0.02
* qPHv8* (w8)	8	92	12	1.8	−1.0	−0.6	0.04	RM6765	0.0013	0.01
Plant height- increasing rate, %										
* qPHR1* (w6/w4)	1	140	70	4	−0.1	−0.03	0.27	RM472	<0.0001	0.09
* qPHR2* (w6/w4)	2	114	30	−2	0.2	−0.1	0.11	RM5363	<0.0001	0.05
(w8/w6)		114	19	−2	−1.0	0.5	0.06	RM5363	0.0228	0.02
* qPHR6.1* (w6/w4)	6	37	15	2	0.1	0.1	0.06	RM204	0.0050	0.02
(w8/w6)		37	16	2	−0.3	−0.2	0.07	RM204	0.0003	0.03
* qPHR6.2* (w6/w4)	6	125	15	−11	1.7	−0.2	0.09	RM5314	0.0156	0.02

QTL, quantitative trait loci; Ch, chromosome; CIM, composite interval mapping; SMA, single marker analysis; ANOVA, analysis of variance.

a Measurements in parenthesis are used to detect the QTL.

b Likelihood ratio (LR) at the peak (cM) position, additive (*a*) and dominance (*d*) effects, and proportion of the phenotypic variance explained by the locus (*R*^2^) were computed by the CIM program based on a subpopulation of 188 (N) F_2_ plants.

c The marker nearest the peak was used to confirm the QTL with the whole population of 480-493 (N) F_2_ plants by MA. The F-test probability level (*P*) and *R*^2^ were estimated by one-way ANOVA.

One and two QTL were associated with tiller numbers (*qTNv1*) and tiller number-increasing rate (*qTNR1* and *5*), respectively, during the vegetative growth phase (Table 5, see Figure S2N). Both *qTNv1* and *qTNR1* were same for the peak position and had the effect-increasing allele from EM93-1. *qTNR5* (*R^2^* = 0.04) contributed less to the phenotypic variance than *qTNR1* (*R^2^* = 0.14) and had the effect-increasing allele from US1.

The aforementioned 49 QTL detected in the subpopulation were all confirmed with the full population, as shown by significant levels (*P* values) from the SMA for the nearest markers (Table 3, Table 4, and Table 5). In general, SMA underestimated the QTL effects (as shown by *R^2^* values), mainly because of distances between the marker and QTL peak positions. These 49 QTL were located on 10 chromosomes (1 to 9 and 12), with 45 (92%) of them collocated as clusters on 14 chromosomal regions (Figure 3). Of the 14 QTL clusters (CL), one encompasses the QTL for crop-mimic (CL5), two contain the QTL for wild traits (CL4.2 and CL7.2), and the remaining 11 have QTL for both wild and crop mimic traits.

### Haplotype variants for QTL cluster regions in U.S. weedy rice

The physical length of haplotypes for the 14 QTL clusters varies from 3.2 to 12.0 Mb. The number of haplotypes varied with the clusters from two to 10 in the sample of 28 U.S. weedy rice lines and from four to 10 in the sample of 14 wild rice lines (Table 6). The correlation between the lengths and haplotype numbers was not significant in the weedy (*r* = 0.38, *P* = 0.18) or wild (*r* = 0.03, *P* = 0.94) rice samples, suggesting that the physical size was not a major contributor to the haplotypic variation. On average, the number of haplotypes was significantly smaller (*t* = -3.4, *P* = 0.002) in the weedy (6.1 ± 2.4) than in the wild (7.5 ± 1.8) rice sample, indicating that U.S. weedy rice is less diverse than wild rice for the QTL-rich regions.

**Table 6:** Summary of haplotypes for the 14 QTL-cluster regions in U.S. weedy rice and wild rice

Cluster		Weedy rice*^c^*				Wild rice*^c^*					
Code*^a^*	Length, Mb*^b^*	N	EM93-1	US1	others	N	EM93-1	US1	others	Share*^d^*	Traits (Table 1) associated with the cluster
CL1.1	5.7 (17.6−23.3)	3	0.00	0.29	0.71	6	0.21	0.29	0.50	3 (100)	SS, SW, HC
CL1.2	3.2 (37.1−40.3)	6	0.00	0.29	0.71	10	0.00	0.00	1.00	1 (17)	TNR, PHR, SN, PH, SD, FT
CL7.1	4.1 (7.0−11.1)	5	0.00	0.36	0.64	7	0.00	0.00	1.00	3 (60)	SD, PC, FT
CL5	7.8 (3.0−10.8)	10	0.00	0.29	0.71	10	0.14	0.00	0.86	1 (10)	TNR, RTN
CL4.1	12.0 (7.9−19.9)	5	0.07	0.29	0.64	6	0.07	0.43	0.50	3 (60)	AN, SS
CL8.2	7.7 (20.2−27.9)	8	0.21	0.21	0.58	8	0.00	0.00	1.00	2 (25)	AN, PH, PHR
CL9	7.0 (3.8−10.8)	7	0.25	0.39	0.36	6	0.00	0.00	1.00	2 (29)	SH, SN
CL7.2	8.2 (21.0−29.3)	6	0.32	0.04	0.64	8	0.00	0.00	1.00	3 (60)	SD, SH
CL3	9.3 (0.5−9.8)	10	0.43	0.04	0.53	10	0.00	0.00	1.00	3 (33)	AN, SS, PHR
CL2	10.3 (19.3−29.6)	8	0.43	0.11	0.46	8	0.07	0.21	0.71	3 (38)	PHR, AN
CL4.2	5.0 (21.9−26.9)	6	0.54	0.18	0.28	8	0.00	0.21	0.89	3 (50)	SD, HC
CL6.1	6.1 (3.2−9.3)	2	0.57	0.43	0.00	6	0.21	0.29	0.50	2 (100)	FT, AN, PHR, LSC, SD, PC
CL8.1	3.7 (2.1−5.8)	6	0.61	0.04	0.35	8	0.00	0.00	1.00	2 (40)	SW, HC
CL6.2	5.2 (22.0−27.2)	3	0.75	0.18	0.07	4	0.29	0.14	0.57	3 (100)	PH, AN, PHR
Mean		6.1	0.30	0.22	0.48	7.5	0.07	0.11	0.82	2.4 (52)	

QTL, quantitative trait loci; SS, seed setting; SW, seed weight; HC, hull color; TNR, tiller number−increasing rate; PHR, plant height−increasing rate; SN, seed number per plant; PH, plant height; SD, seed dormancy; FT, flowering time; RTN, number of reproductive tillers/plant; AN, awn; SH, seed shattering; LSC, leaf sheath color; and PC, pericarp color.

a Refer to Figure 3 for map/chromosomal positions of clusters (CL).

b Physical length (interval) determined by the flanking marker positions (Mb) on the reference genome sequence (Gramene 2012).

c Number of haplotypes (N) and frequencies of the EM93-1- and US1-like haplotypes, and other haplotypes different from the EM93-1- or US1-like in the weedy and wild rice samples.

d Number (percentile) of haplotypes shared between the weedy and wild rice samples.

Shared haplotypes between the weedy and wild rice samples ranged from 10 to 100%, with the average being 52%, across the 14 cluster regions (Table 6). The EM93-1−like haplotypes were present in 10 of the 14 QTL regions and accounted for 30% of the haplotypes on average in the weedy rice sample. In contrast, the EM93-1−like haplotypes were present only in six of the 14 regions and represent 7% of the haplotypes in the wild rice sample. Of the four QTL cluster regions (CL1.1, 1.2, 5, and 7.1) absent of the EM93-1-like haplotypes, CL1.1 and CL1.2 link on an interval of ~40 cM containing *sd1* and CL7.1 contains *Rc* (Figure 3). The absence of the EM93-1−like haplotypes indicates that U.S. red rice populations have maintained relatively large blocks of linkage disequilibrium (LD) to cover the wild-type alleles *Sd1* and *Rc* since the founder genotypes were introduced into the new continent. The US1-like haplotypes were present in all the 14 QTL regions and account for 22% of the haplotypes in the sample of U.S. weedy rice. In contrast, the US1-like haplotypes were present in six of the 14 QTL regions and represent 11% of the haplotypes in the wild rice sample.

**Figure 4:**
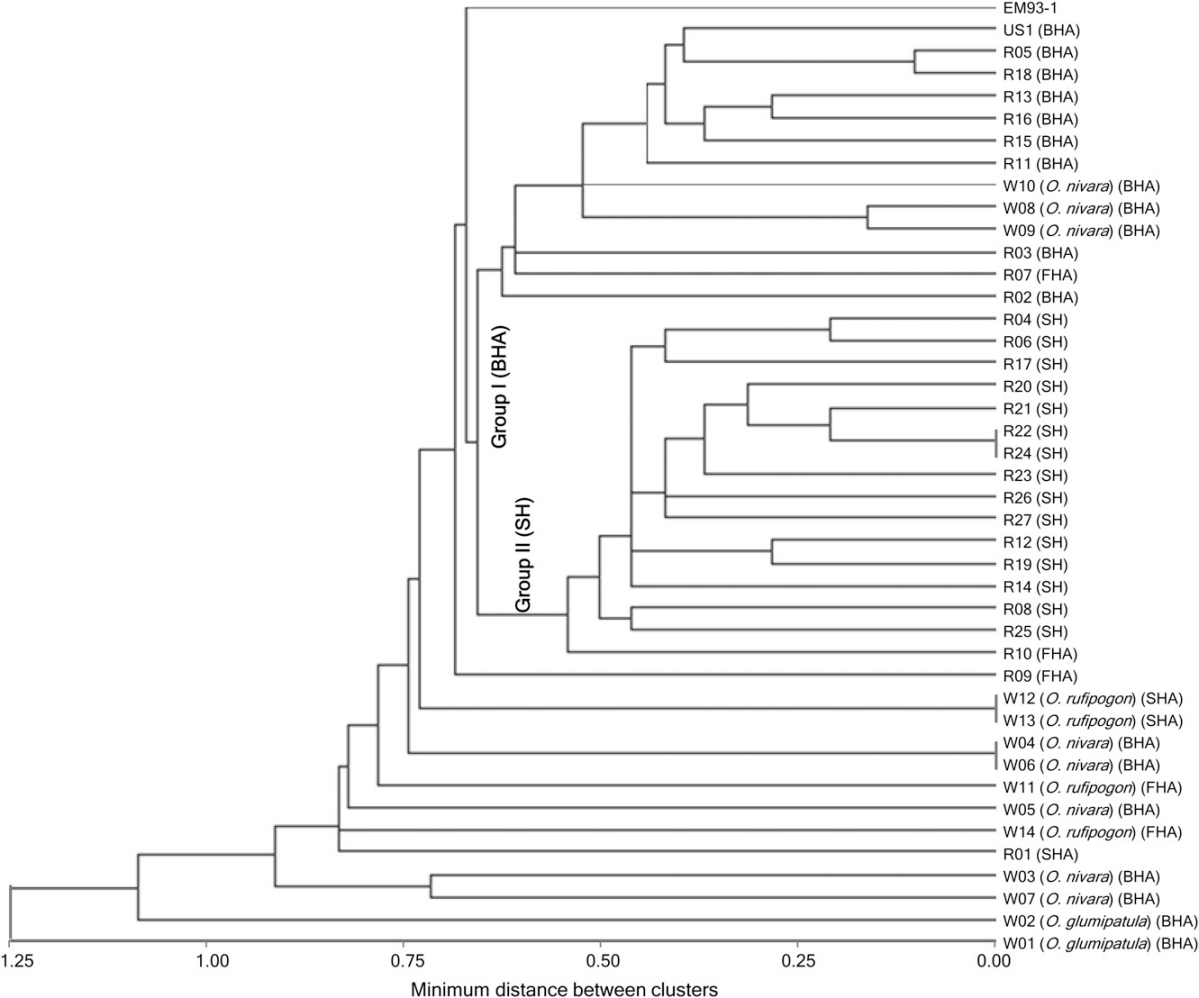
Phylogenetic relationship of selected U.S. weedy and wild rice lines. The dendrogram is developed based on the haplotypic data for the 14 QTL cluster regions (Figure 3). The weedy red (US1 and R01-27) and wild (W01-14) rice lines belong to the four seed morphological types (Figure S1): black-hull awned (BHA), furrow-hull awned (FHA), straw-hull awned (SHA), and straw-hull anwless (SH).

Based on the haplotype data for the 14 QTL cluster regions, the 28 weedy rice lines were separated from most (9/14) of the wild rice lines and 26 of the 28 clustered into two groups (Figure 4). Group I includes 10 (nine BHA and one FHA) weedy and 3 BHA wild rice lines, while Group II consists of 16 (15 SH and 1 FHA) weedy rice. The other two red rice lines, R01 (PI-506229, SHA) from California and R09 (PI-653420, FHA) from Louisiana, appeared to be close to FHA or SHA wild rice.

## Discussion

### Trait correlations and QTL clusters

This research confirmed the previous observation that wild traits are interrelated phenotypically (Gu *et al.* 2005c) and also revealed that the phenotypic correlation is common between wild and crop-mimic traits in weedy rice. For example, the characters seed shattering, awn presence, red/brown pericarp color, purple leaf sheath color, and seed dormancy were positively or negatively correlated with several of the eight crop mimic traits in the large F_2_ population (Table 2). Natural selection tended to assemble the wild characters, also known as “adaptive syndromes,” to make a weed plant weedy (Harlan 1965; Oka 1988). The natural selection in agro-ecosystems is expected, to some extent, to impact weed phenotypes for multiple crop-mimic traits, including plant height- and tiller number-increasing rates that contribute to early plant competitiveness. The plant height and tiller number traits differed in dynamic pattern during the vegetative growth phase (Figure 2, A and D) and were correlated with the final plant height and number of reproductive tillers (Table 2). However, the correlations accounted for only <10% of the phenotypic variances, suggesting that there are additional genes imparting the early plant competitiveness. QTL analysis in the previous and this research provided valuable insight into genetic basis and genomic structures underlying individual wild and crop-mimic traits and trait correlations.

More QTL were detected for wild and crop-mimic traits in this than in the reported research on weedy rice (Bres-Patry *et al.* 2001; Gu *et al.* 2005a; Jing *et al.* 2008; Subudhi *et al.* 2012; Thurber *et al.* 2013). In addition to the difference in the parental combination of mapping population from the previous research, a larger population size, the greenhouse condition, and more trait measurements used in this research must have also contributed to the QTL detection. It was unforeseen that greater than 90% of the QTL collocated as clusters on 14 genomic regions. The QTL distribution patterns on the genome explain the observed trait correlations. In addition, more QTL clusters detected in this research indicate that “a small number of chromosomal segments” (Bres-Patry *et al.* 2001) is insufficient to explain the conspecific weed-crop differentiation for adaptive or domestication-related traits.

Both correlated and uncorrelated traits may have their QTL collocated on the same genomic segments. It was estimated that correlated traits had more collocated QTL or shared QTL clusters (33% on average) than uncorrelated traits (11%) (Gardner and Latta 2007). Of the 49 pairs of trait correlation listed in Table 2, 22 (45%) had one or more shared QTL clusters, which is higher than the previous estimation. On the other hand, of the 70 QTL pairs in the 14 clusters, 45 (64%) displayed trait correlations (29 positive and 16 negative), and the remaining 21 (36%) did not correlate phenotypically in the mapping population (see Table S3). For example, *qSD1.2* was clustered with *qSN1*, *qPH1*, *qPHR1*, and *qTNR1* in cluster CL1.2, although SD was not correlated with any of the SN, PH, PHR, and TNR traits. A higher level of the correspondence between trait correlations and QTL clusters was observed for the 5 wild traits (SH, SD, AN, HC, and PC) in our previous research, most likely because their heritability levels were relatively high (Gu *et al.* 2005a). Genetic background effects also interfere with the expression of trait correlation or clustered QTL. For example, the correlation between seed dormancy and plant height and their QTL clusters *qSD1.2*/*qPH1* and *qSD7.2*/*qPH7* were detected only in advanced backcross populations (Ye *et al.* 2013). Additional data can be found at Table S4, Table S5, and Table S6.

There is evidence that trait correlations in weedy rice could arise from pleiotropic effects of single genes, or the linkage between genes for the correlated traits, or both pleiotropy and linkage, although these two mechanisms are often confounded at the QTL level. Cluster CL7.1 consists of *qPC7* for pericarp color, *qSD7.1* for seed dormancy, and *qFT7* for flowering time. Both *qPC7* and *qSD7.1* are underlain by *Rc* and share the same functional nucleotide polymorphism or FNP (Gu *et al.* 2011), whereas *qFT7* has been finely mapped to the position a few centimorgan (~6 Mb) from *Rc* and genetically isolated from *qSD7.1* (Gu and Foley 2007 and unpublished data). Cluster CL1.2 encompasses eight QTL for plant height, seed dormancy, and other growth- related traits and also the known gene *sd1* encoding gibberellin 20-oxidase. Fine mapping, gene expression analysis, and gibberellin induction analysis indicated that weedy rice carries the wild allele at *sd1* to promote seedling/plant growth and also suggested that the mutant allele *sd1* from the parent EM93-1 most likely has a pleiotropic effect on enhanced seed dormancy (Ye *et al.* 2013). Recent research identified additional FNPs at *sd1* (Asano *et al.* 2011) and new plant height QTL (Kovi *et al.* 2011) in the CL1.2 region, suggesting that a QTL cluster could be underlain by multiple molecular genetic mechanisms.

### Implications of haplotypic variants for the multi-QTL regions

The haplotype analysis revealed that U.S. weedy red rice populations are highly differentiated in genomic regions containing multiple QTL responsible for adaptation and competitiveness. The haplotype diversity suggests that the wild and crop-mimic traits segregating in the mapping population may also differentiate among ecotypes or populations of weedy rice. About 50% of the haplotypes in the 28 weedy rice lines were also present in the 14 lines of the AA-genome wild rice (*O*. spp.). These shared wild-like haplotypes were presumably derived from introduced founder genotypes, as there was no wild rice in North America, and have been maintained by LD and natural selection during the evolution of red rice populations. Some of the LD blocks are >10 Mb in length (*e.g.*, CL2 and 4.1) or 100% wild-like (*e.g.*, CL1.1, 6.1, and 6.2). The parental line EM93-1 was developed by a hybridization of two semi-dwarf *indica* cultivars from China (Gu *et al.* 2004b), which have no direct pedigree relationship with the U.S. weedy rice lines. The presence of EM93-1−like haplotypes in the weedy rice sample could not be a random event, for the frequency (30%) is much higher than that (7%) in the wild rice sample. Therefore, the haplotypes other than the wild-like ones in U.S. red rice populations were most likely incorporated from old cultivars. The haplotypes like the weedy rice parent US1 accounted for ∼20% of the haplotypes in the 28 red rice lines. This is indicative that the current U.S. weedy rice populations may originate from multiple founder genotypes. Further research could extend the haplotype analysis to old cultivars/landraces, including those from *aus* and *indica* ecotypes (Reagon *et al.* 2010), to help identify the founder genotypes of U.S. weedy rice.

U.S. weedy rice populations are often classified based on HC and AN presence/absence in practice. This simple classification system has been shown in a good agreement with the classification based on genomic patterns of randomly selected DNA markers (Reagon *et al.* 2010) and the haplotype patterns for the QTL cluster regions (Figure 4). This research identified three HC and six AN QTL and these nine loci distribute on nine different genomic segments of six chromosomes (Figure 3). Thurber *et al.* (2013) detected two HC and three AN QTL from U.S. weedy rice, with two of the AN loci on the two additional chromosomes. Thus, phenotypic variation for the HC and AN morphologies is regulated by at least 11 functionally differentiated loci scattered on eight chromosomes. The genome distribution pattern of the multiple QTL explains the good agreement between the morphological and marker-based classification systems. It is expected that the morphological classification system will continue to be useful because of the genome distribution of HC and AN loci and their linkage with genes for the other adaptive traits in the clusters.

## Supplementary Material

Supporting Information

## References

[bib1] Asano K. Yamasaki M. Takuno S. Katagiri S. Ito T., 2011 Artificial selection for a green revolution gene during *japonica* rice domestication. Proc. Natl. Acad. Sci. USA 108: 11034–11039 21646530 10.1073/pnas.1019490108 PMC3131315

[bib2] Baker H. G., 1974 The evolution of weeds. Annu. Rev. Ecol. Syst. 5: 1–24

[bib3] Booth B. D. Murphy S. D. Swanton C. J., 2003 Weed Ecology in Natural and Agricultural Systems. CABI, Wallingford, UK

[bib4] Bres-Patry C. Lorieux M. Clement G. Bangratz M. Ghesquiere A., 2001 Heredity and genetic mapping of domestication-related traits in a temperate *japonica* weedy rice. Theor. Appl. Genet. 102: 118–126

[bib5] Cao Q. Lu B. R. Hui X. Jun R. Sala F., 2006 Genetic diversity and origin of weedy rice (*Oryza sativa* f. *spontanea*) populations found in north-eastern China revealed by simple sequence repeat (SSR) markers. Ann. Bot. (Lond.) 98: 1241–1252 10.1093/aob/mcl210 PMC3292271 17056615

[bib6] Delouche J. C. Burgos N. R. Gealy D. R. de San Martin G. Z. Labrada R., 2007 *Weedy Rices- Origin*, *Biology*, *Ecology and Control*. FAO, Rome, Italy

[bib7] Ellstrand N. C. Prentice H. C. Hancock J. F., 1999 Gene flow and introgression from domesticated plants into their wild relatives. Annu. Rev. Ecol. Syst. 30: 539–563

[bib8] Furukawa T. Maekawa M. Oki T. Suda I. Iida S., 2007 The Rc and Rd genes are involved in proanthocyanidin synthesis in rice pericarp. Plant J. 49: 91–102 17163879 10.1111/j.1365-313X.2006.02958.x

[bib9] Gardner K. M. Latta R. G., 2007 Shared quantitative traits loci underlying the genetic correlation between continuous traits. Mol. Ecol. 16: 4195–4209 17850272 10.1111/j.1365-294X.2007.03499.x

[bib10] Gramene, 2012 *Oryza sativa* genome sequence (Gramene release 36). Available at: 2012 at http://www.gramene.org/Oryza_sativa/Info/Index Accessed: December 2012.

[bib11] Gu X.-Y. Kianian S. F. Foley M. E., 2004 a Multiple loci and epistases control genetic variation for seed dormancy in weedy rice (*Oryza sativa*). Genetics 166: 1503–1516 15082564 10.1534/genetics.166.3.1503 PMC1470771

[bib12] Gu X.-Y. Foley M. E. Chen Z., 2004 b A set of three genes regulates photoperiodic responses of flowering in rice (*Oryza sativa*). Genetica 122: 127–140 15609572 10.1023/b:gene.0000041003.12834.41

[bib13] Gu X.-Y. Kianian S. F. Hareland G. A. Hoffer B. L. Foley M. E., 2005 a Genetic analysis of adaptive syndromes interrelated with seed dormancy in weedy rice (*Oryza sativa*). Theor. Appl. Genet. 110: 1108–1118 15782297 10.1007/s00122-005-1939-2

[bib14] Gu X.-Y. Kianian S. F. Foley M. E., 2005 b Phenotypic selection for dormancy introduced a set of adaptive haplotypes from weedy into cultivated rice. Genetics 171: 695–704 15972459 10.1534/genetics.105.043612 PMC1456781

[bib15] Gu X.-Y. Kianian S. F. Foley M. E., 2005 c Dormancy imposed by covering tissues interrelated with seed shattering and morphological characteristics in weedy rice (*Oryza sativa* L.). Crop Sci. 45: 948–955

[bib16] Gu X.-Y. Foley M. E., 2007 Epistatic interactions of three loci regulate flowering time under short and long daylengths in a backcross population of rice. Theor. Appl. Genet. 114: 745–754 17171390 10.1007/s00122-006-0475-z

[bib17] Gu X.-Y. Foley M. E. Horvath D. P. Anderson J. V. Feng J., 2011 Association between seed dormancy and pericarp color is controlled by a pleiotropic gene that regulates abscisic acid and flavonoid synthesis in weedy red rice. Genetics 189: 1515–1524 21954164 10.1534/genetics.111.131169 PMC3241415

[bib18] Harlan J. R., 1965 The possible role of weed races in the evolution of cultivated plants. Euphytica 14: 173–176

[bib19] Ishikawa R. Toki N. Imai K. Sato Y. Yamagishi H., 2005 Origin of weedy rice grown in Bhutan and the force of genetic diversity. Genet. Resour. Crop Evol. 52: 395–403

[bib20] Jing W. Jiang L. Zhang W. Zhai H. Wan J., 2008 Mapping QTL for seed dormancy in weedy rice. Acta Agron. Sin. 34: 737–742

[bib21] Konishi S. Izawa T. Lin S. Y. Ebana K. Fukuta Y., 2006 An SNP caused loss of seed shattering during rice domestication. Science 312: 1392–1396 16614172 10.1126/science.1126410

[bib22] Kovi M. R. Zhang Y. Yu S. Yang G. Yan W., 2011 Candidacy of a chitin-inducible gibberellins-responsive gene for a major locus affecting plant height in rice that is closely linked to Green Revolution gene *sd1*. Theor. Appl. Genet. 123: 705–714 21637999 10.1007/s00122-011-1620-x

[bib23] Kush G. S. Brar D. S., 2002 Rice, pp. 1–41 in Evolution and Adaptation of Cereal Crops, edited by Chopra V. L. Prakash S. Science Publishers Inc., Enfield, NH

[bib24] Li C. Zhou A. Sang T., 2006 Rice domestication by reducing shattering. Science 311: 1936–1939 16527928 10.1126/science.1123604

[bib25] Lincoln S. Daly M. Lander E., 1992 Constructing Genetic Maps with MAPMAKER/EXP 3.0. 3rd ed. Whitehead Institute, Cambridge, MA

[bib26] Londo J. P. Schaal B. A., 2007 Origins and population genetics of weedy red rice in the USA. Mol. Ecol. 16: 4523–4535 17887969 10.1111/j.1365-294X.2007.03489.x

[bib27] McCouch S. R. Teytelman L. Xu Y. B. Lobos K. B. Clare K., 2002 Development and mapping of 2240 new SSR markers for rice (*Oryza sativa* L.). DNA Res. 9: 199–207 12597276 10.1093/dnares/9.6.199

[bib28] Noldin J. A. Chandler J. M. McCauley G. N., 1999 Red rice (*Oryza sativa*) Biology. I. Characterization of red rice ecotypes. Weed Technol. 13: 12–18

[bib29] Oka H. I., 1988 Origin of Cultivated Rice. Japan Science Society Press, Tokyo

[bib30] Reagon M. Thurber C. S. Gross B. L. Olsen K. M. Jia Y., 2010 Genomic patterns of nucleotide diversity in parallel populations of U.S. weedy rice. BMC Evol. Biol. 10: 180 20550656 10.1186/1471-2148-10-180 PMC2898691

[bib31] Reagon M. Thurber C. S. Olsen K. M. Jia Y. Caicedo A. L., 2011 The long and the short of it: *SD1* polymorphism and the evolution of growth trait divergence in U.S. weedy rice. Mol. Ecol. 20: 3743–3756 21854475 10.1111/j.1365-294X.2011.05216.x

[bib32] SAS Institute, 2011 SAS/STAT 9.3 User’s Guide. SAS Institute Inc, Cary, NC

[bib33] Subudhi P. K. Parco A. Singh P. K. DeLeon T. Karan R., 2012 Genetic architecture of seed dormancy in U.S. weedy rice in different genetics backgrounds. Crop Sci. 52: 2564–2575

[bib34] Suh H. S. Sato Y. I. Morishima H., 1997 Genetic characterization of weedy rice (*Oryza sativa* L.) based on morphophysiology, isozymes and RAPD markers. Theor. Appl. Genet. 94: 316–321

[bib35] Sweeney M. T. Thomson M. J. Pfeil B. E. McCouch S. R., 2006 Caught red-handed: *Rc* encodes a basic helix-loop-helix protein conditioning red pericarp in rice. Plant Cell 18: 283–294 16399804 10.1105/tpc.105.038430 PMC1356539

[bib36] Tang L. H. Morishima H., 1997 Genetic characterization of weedy rices and the inference on their origins. Breed. Sci. 47: 153–160

[bib37] Thurber C. S. Reagon M. Gross B. L. Olsen K. M. Jia Y., 2010 Molecular evolution of shattering loci in U.S. weedy rice. Mol. Ecol. 19: 3271–3284 20584132 10.1111/j.1365-294X.2010.04708.x PMC2988683

[bib38] Thurber C. S. Jia M. H. Jia Y. Caicedo A. L., 2013 Similar traits, different genes? Examining convergent evolution in related weedy rice populations. Mol. Ecol. 22: 685–698 23205731 10.1111/mec.12147

[bib39] Wang, S., C. J. Basten, and Z. B. Zeng, 2006 *QTL Cartographer 2.5* Department of Statistics, North Carolina State University, Raleigh, NC, http://statgen.ncsu.edu/qtlcart/WQTLCart.htm

[bib40] Ye H. Foley M. E. Gu X.-Y., 2010 New seed dormancy loci detected from weedy rice-derived advanced populations with major QTL alleles removed from the background. Plant Sci. 179: 612–619

[bib41] Ye H. Beighley D. H. Feng J. Gu X.-Y., 2013 Genetic and physiological characterization of two clusters of quantitative trait loci associated with seed dormancy and plant height in rice. G3 (Bethesda) 3: 323–331 23390608 10.1534/g3.112.005041 PMC3564992

[bib42] Yoshida S. Forno D. A. Cock J. H. Gomez K. A., 1976 Laboratory Manual For Physiological Studies Of Rice. 3rd ed. IRRI, Manila, The Philippines

