# Supplementary material for: Quantitative Trait Locus and Haplotype Analyses of Wild and Crop-Mimic Traits in U.S. Weedy Rice
Source: G3 (Bethesda). 2013 Jun 1;3(6):1049–59. doi: 10.1534/g3.113.006395 (PMC3689802; doi:10.1534/g3.113.006395)
Supplement: Supporting Information [file supp_3_6_1049__index.html]

Quantitative Trait Locus and Haplotype Analyses of Wild and Crop-Mimic Traits in U.S. Weedy Rice — Supporting Information 

# Quantitative Trait Locus and Haplotype Analyses of Wild and Crop-Mimic Traits in U.S. Weedy Rice

## Supporting Information for Mispan *et al.*, 2013

**Files in this Data Supplement:**

- Supporting Information - Figures S1-S2 and Tables S1-S6 (PDF, 396 KB)
- Figure S1 - Seed morphologies of U.S. weedy red rice and wild rice (PDF, 236 KB)
- Figure S2 - Genome wide scan for QTL associated with wild and crop-mimic traits in the F2 EM93-1/US1 population (PDF, 148 KB)
- Table S1 - List of information on U.S. weedy red rice and wild rice lines used for this research (PDF, 82 KB)
- Table S2 - List of markers with a segregation ratio deviated from the expected 1:2:1 in the F2 EM93-1/US1 population (PDF, 79 KB)
- Table S3 - Summary of observed and predicted pairs of trait correlation in the F2 population (PDF, 70 KB)
- Table S4 - Phenotypic data for wild and crop-mimic traits segregating in the F2 EM93-1/US1 population (.xlsx, 122 KB)
- Table S5 - Marker genotyping data for individuals in the F2 EM93-1/US1 population (.xlsx, 251 KB)
- Table S6 - Genotyping data for markers used to define haplotypes for the 14 QTL cluster regions in 28 U.S. weedy and 14 wild rice lines (.xlsx, 16 KB)
