# Supplementary material for: Quantitative Trait Locus and Haplotype Analyses of Wild and Crop-Mimic Traits in U.S. Weedy Rice
Source: G3 (Bethesda). 2013 Jun 1;3(6):1049–59. doi: 10.1534/g3.113.006395 (PMC3689802; doi:10.1534/g3.113.006395)
Supplement: Supporting Information [file supp_g3.113.006395_006395SI.pdf]

G3: Genes | Genomes | Genetics

Quantitative Trait Locus and Haplotype Analyses of Wild and Crop-Mimic Traits in U.S. Weedy Rice

Muhamad S. Mispan, Lihua Zhang, Jiuhuan Feng, and Xing-You Gu

Plant Science Department, South Dakota State University, Brookings, SD 57007, USA

DOI: 10.1534/g3.113.006395

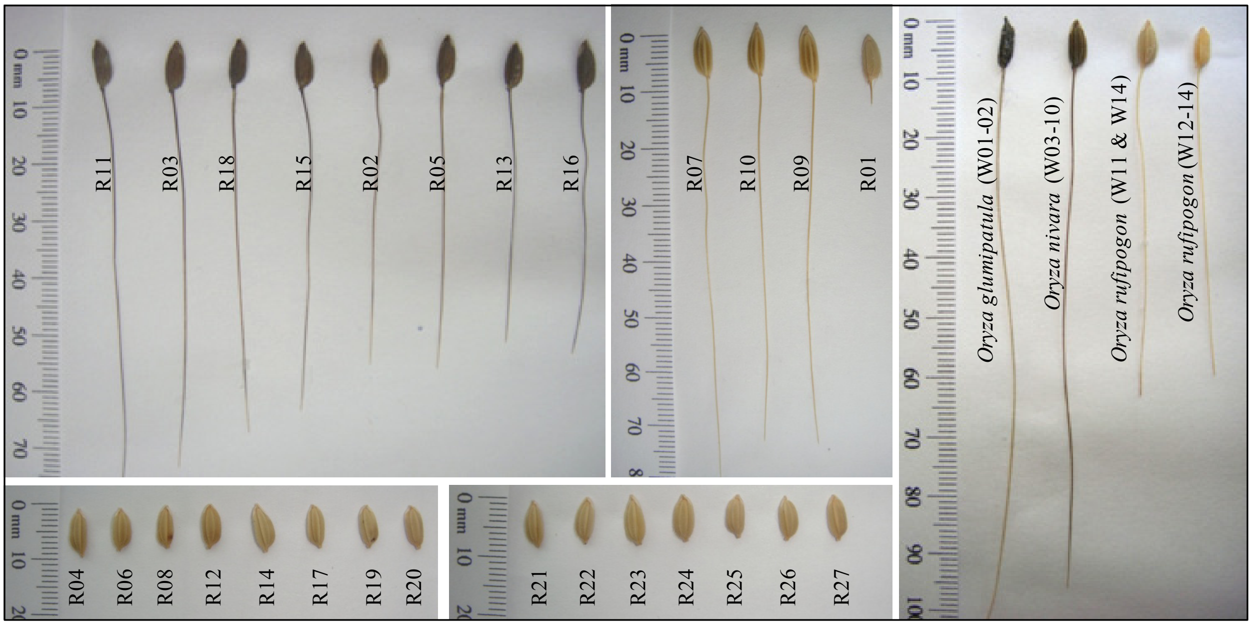

Figure S1 Seed morphologies of U.S. weedy red rice and wild rice. Refer to Table S1 for additional information about the red (R01-R27) and wild (W01-W14) rice.

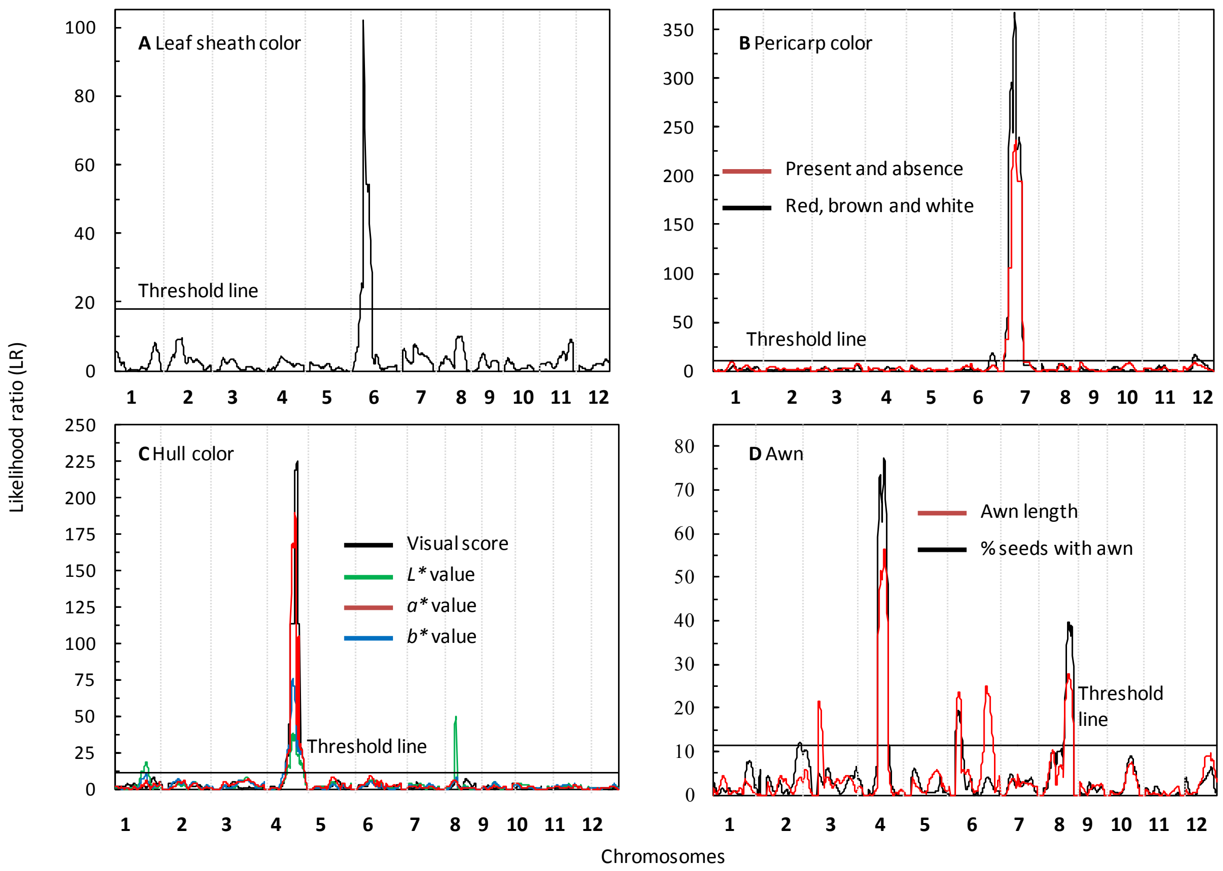

Figure S2 Genome wide scan for QTL associated with wild and crop-mimic traits in the F_2 EM93-1/US1 population. (A) Leaf sheath color (LSC); (b) pericarp color (PC), (c) hull color (HC), (d) awn (AN), (e) seed shattering (SH), (f) seed dormancy (SD), (g) flowering time (FT), (h) reproductive tiller numbers/plant (RTN), (i) plant height (PH), (j) seed weight (SW), (k) seed numbers/plant (SN) and seed setting percentage (SSP), (l) tiller number at vegetative stage (TNv) and tiller number-increasing rates (TNR) for week 6/week 4 (w6/w4) and w8/w6, (m) plant height at vegetative stage (PHv), and (n) plant height increasing rates (PHR) for for week 6/week 4 (w6/w4) and w8/w6.

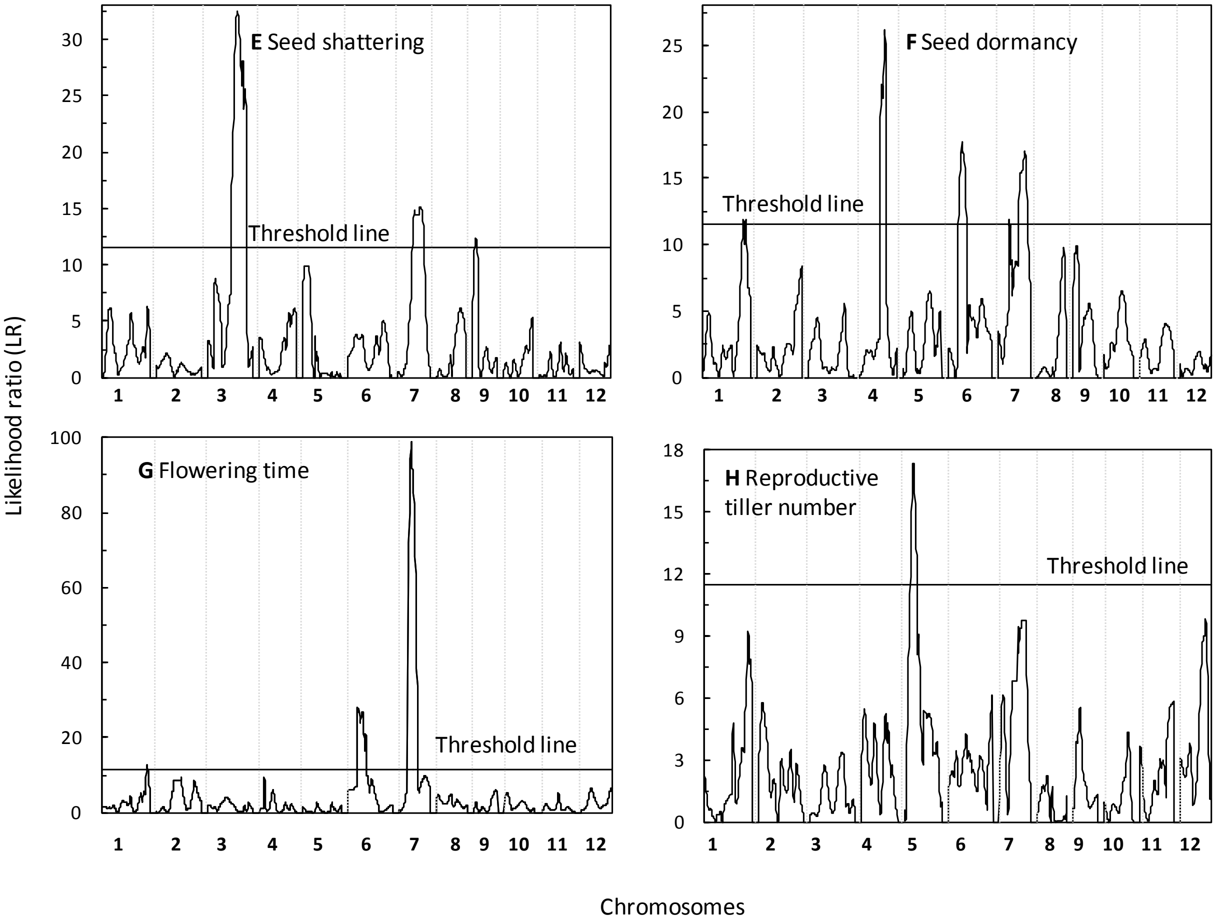

Figure S2 Continued.

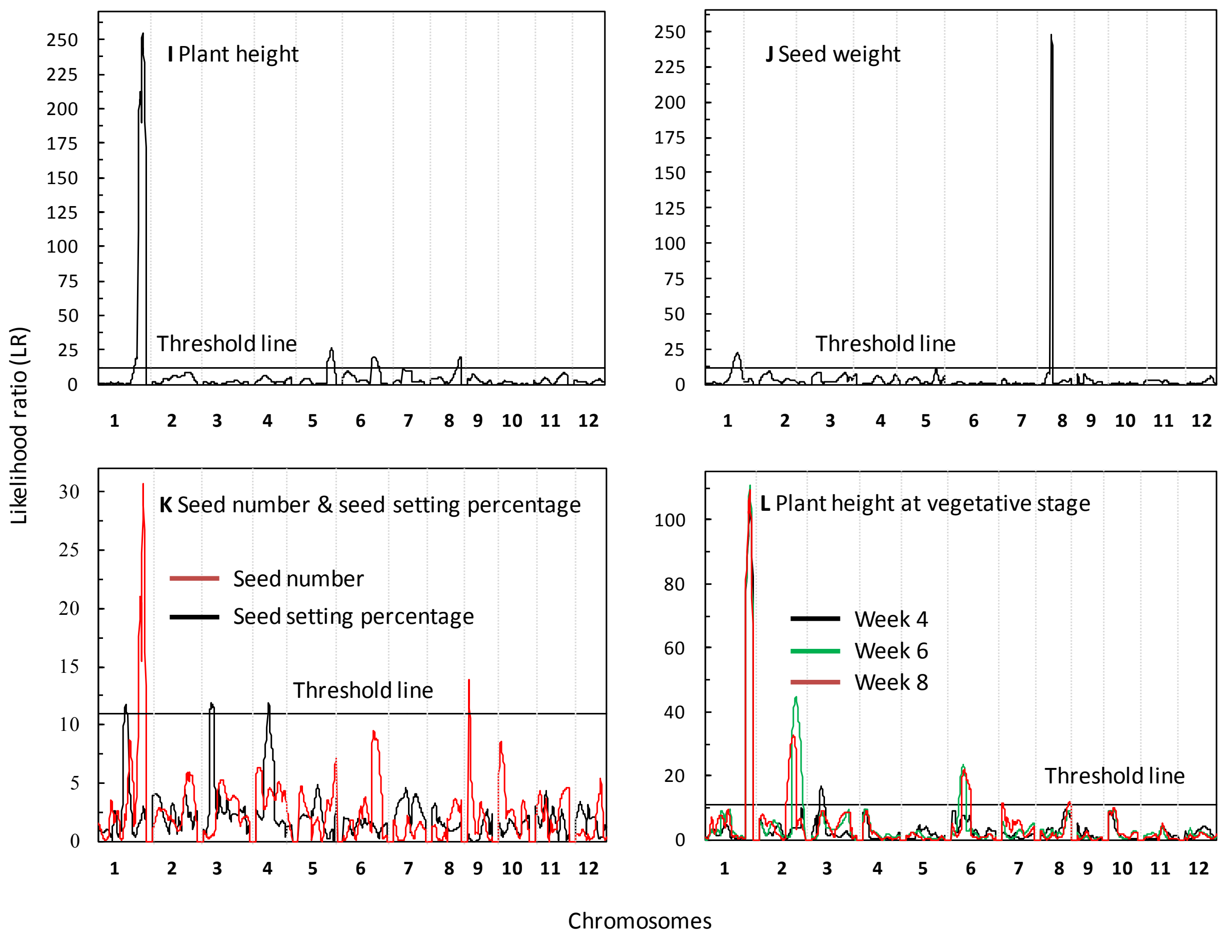

Figure S2 Continued.

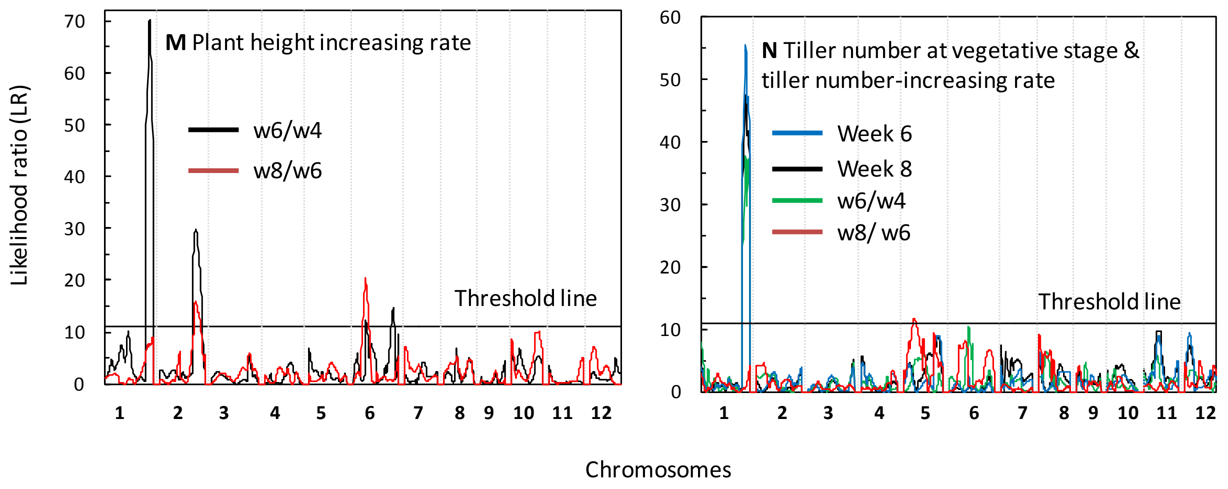

Figure S2 Continued.

Table S1 List of information on U.S. weedy red rice and wild rice lines used for this research

Code[a]	PI #[b]	Taxon[b]	Origin[b]	Phenotypes[c]				
				Hull color	Pericarp color	Awn (length in mm)	Height (cm)	Days to flowering
R01	PI-506229	*O. sativa*	California	Straw	Red	Short (<5)	66	95
R02	PI-653412	*O. sativa*	Arkansas	Black	Red	Long (35)	86	70
R03	PI-653413	*O. sativa*	Arkansas	Black	Red	Long (55)	118	97
R04	PI-653414	*O. sativa*	Arkansas	Straw	Red	None	100	86
R05	PI-653415	*O. sativa*	Arkansas	Black	Red	Long (45)	83	117
R06	PI-653416	*O. sativa*	Mississippi	Straw	Red	None	104	131
R07	PI-653417	*O. sativa*	Arkansas	Furrowed	Red	Long (60)	98	97
R08	PI-653418	*O. sativa*	Mississippi	Straw	Red	None	100	131
R09	PI-653420	*O. sativa*	Louisiana	Furrowed	Red	Long (60)	102	100
R10	PI-653421	*O. sativa*	Mississippi	Furrowed	Red	Long (55)	103	100
R11	PI-653422	*O. sativa*	Arkansas	Black	Red	Long (55)	120	105
R12	PI-653423	*O. sativa*	Arkansas	Straw	Red	None	104	86
R13	PI-653425	*O. sativa*	Arkansas	Black	Red	Long (30)	119	100
R14	PI-653426	*O. sativa*	Missouri	Straw	Red	None	99	78
R15	PI-653427	*O. sativa*	Arkansas	Black	Red	Long (40)	110	96
R16	PI-653428	*O. sativa*	Arkansas	Black	Red	Long (25)	128	96
R17	PI-653429	*O. sativa*	Arkansas	Straw	Red	None	97	80
R18	PI-653430	*O. sativa*	Arkansas	Black	Red	Long (55)	83	117
R19	PI-653431	*O. sativa*	Arkansas	Straw	Red	None	105	70
R20	PI-653432	*O. sativa*	Missouri	Straw	Red	None	105	80
R21	PI-653433	*O. sativa*	Arkansas	Straw	Red	None	90	90
R22	PI-653434	*O. sativa*	Arkansas	Straw	Red	None	97	90
R23	PI-653435	*O. sativa*	Arkansas	Straw	Red	None	90	79
R24	PI-653436	*O. sativa*	Arkansas	Straw	Red	None	94	79

R25	PI-653437	*O. sativa*	Louisiana	Straw	Red	None	62	70
R26	PI-653438	*O. sativa*	Mississippi	Straw	Red	None	72	76
R27	PI-653439	*O. sativa*	Louisiana	Straw	Red	None	75	80
Wild rice								
W01	PI-590413	*O. glumipatula*	N/A	Black	Red	Long (95)	127	N/A
W02	PI-527364	*O. glumipatula*	Brazil	Black	Red	Long (95)	132	N/A
W03	PI-590426	*O. nivara*	N/A	Black	Red	Long (85)	78	N/A
W04	PI-590404	*O. nivara*	India	Black	Red	Long (85)	90	N/A
W05	PI-590409	*O. nivara*	India	Black	Red	Long (85)	92	N/A
W06	PI-590411	*O. nivara*	India	Black	Red	Long (85)	90	N/A
W07	GSOR-311698	*O. nivara*	India	Black	Red	Long (85)	105	N/A
W08	PI-590425	*O. nivara*	Myanmar	Black	Red	Long (80)	85	N/A
W09	GSOR-311699	*O. nivara*	Myanmar	Black	Red	Long (80)	127	N/A
W10	PI-590410	*O. nivara*	N/A	Black	Red	Long (80)	132	N/A
W11	PI-590418	*O. rufipogon*	Myanmar	Furrowed	Red	Long (55)	148	N/A
W12	PI-590422	*O. rufipogon*	Myanmar	Straw	White	Long (55)	93	N/A
W13	GSOR-311703	*O. rufipogon*	Myanmar	Straw	White	Long (55)	105	N/A
W14	PI-590417	*O. rufipogon*	Taiwan	Furrowed	Red	Long (55)	142	N/A

[a] Coded for citation in this research (*e.g.,* Fig. 4).

[b] Information from the National Small Grains Collection (NSGC), USDA-ARS.

[c] Observed for plants grown in the greenhouse after the introduction from NSGC. N/A, not available.

Table S2 List of markers with a segregation ratio deviated from the expected 1:2:1 in the F_2 EM93-1/US1 population

Markers	Chr.	Position	Genotypic frequency			Fitness test (χ^2 value)*	Allelic frequency	
			EM93-1-like	Heterozygote	US1-like		EM93-1-like	US1-like
RM3740	1	22.8	0.33	0.49	0.18	8.43	0.57	0.43
RM84	1	30.9	0.32	0.51	0.18	7.78	0.57	0.43
RM283	1	41.2	0.31	0.52	0.17	7.95	0.57	0.43
RM7479	7	14.0	0.35	0.47	0.18	12.12	0.59	0.41
RM1253	7	29.4	0.29	0.54	0.16	7.49	0.56	0.44
RID12	7	34.0	0.34	0.50	0.16	12.30	0.59	0.41
RM6018	7	38.6	0.32	0.53	0.15	10.76	0.58	0.42
RM3635	7	40.2	0.31	0.52	0.16	8.68	0.57	0.43
RM3755	7	43.2	0.30	0.52	0.18	6.47	0.56	0.44
RM346	7	62.6	0.26	0.59	0.16	8.89	0.55	0.45
RM6403	7	71.1	0.31	0.53	0.15	10.34	0.58	0.42
RM38	8	10.3	0.12	0.54	0.34	18.93	0.39	0.61
RM3778	8	23.2	0.06	0.60	0.34	35.35	0.36	0.64
RM6208	8	40.2	0.01	0.48	0.51	96.20	0.25	0.75
RM3395	8	48.9	0.03	0.52	0.45	68.43	0.29	0.71
RM404	8	51.6	0.03	0.53	0.44	62.21	0.30	0.70
RM515	8	65.8	0.10	0.53	0.37	29.53	0.36	0.64
RM5515	9	8.7	0.16	0.55	0.29	8.37	0.43	0.57
RM296	9	21.5	0.17	0.55	0.28	6.41	0.44	0.56
RM6839	9	31.2	0.16	0.52	0.31	8.68	0.43	0.57
RM239	10	24.0	0.18	0.53	0.30	6.16	0.44	0.56
RM20	11	0	0.27	0.60	0.13	14.09	0.57	0.43
RM167	11	18.0	0.27	0.56	0.17	6.51	0.55	0.45
RM7283	11	37.2	0.23	0.61	0.16	11.18	0.53	0.47
RM7003	12	32.6	0.23	0.60	0.16	9.48	0.53	0.47

***** Chi-square value at *P*=0.05 is 5.99.

Table S3 Summary of observed and predicted pairs of trait correlation in the F_2 population

Cluster[a]	No. of QTL[b]	Expected QTL pairs[b]	Observed trait pairs[c]	Predicted trait pairs with[d]	
				positive corr.	negative corr.
CL1.1	3	3	1	1	2
CL1.2	8	28	16	13	15
CL2	3	3	2	1	2
CL3	3	3	2	3	0
CL4.1	2	1	1	1	0
CL4.2	2	1	1	1	0
CL5	2	1	1	1	0
CL6.1	6	15	10	7	8
CL6.2	4	6	4	2	4
CL7.1	3	3	3	3	0
CL7.2	2	1	1	1	0
CL8.1	2	1	0	1	0
CL8.2	3	3	2	3	0
CL9	2	1	1	1	0
Total[e]	45	70	45 (64%)	39 (56%)	31 (44%)

[a] Refer to Fig. 3 for map positions.

[b] Number of QTL and expected QTL pairs in the cluster.

[c] Observed pairs of trait correlation (Table 2).

[d] Predicted based on signs (+/-) of QTL additive effect (*a*) values (Tables 3-5): positive correlation, both QTLs had plus or minus *a*; negative correlation, one QTL had plus while the other had minus *a*.

[e] Percentages are based on the number of expected QTL pairs[b].

Tables S4-S6 are available for download at http://www.g3journal.org/lookup/suppl/doi:10.1534/g3.113.006395/-/DC1.

Table S4 Phenotypic data for wild and crop-mimic traits segregating in the F2 EM93-1/US1 population. Refer to Table 1 for trait abbreviation.

Table S5 Marker genotyping data for individuals in the F2 EM93-1/US1 population. Marker genotypes: 0=EM93-1-like, 1=heterozygous, 2=US1-like homozygous, dot=missing.

Table S6 Genotyping data for markers used to define haplotypes for the 14 QTL cluster regions in 28 U.S. weedy and 14 wild rice lines.
