# Supplementary material for: Quantitative Trait Locus and Haplotype Analyses of Wild and Crop-Mimic Traits in U.S. Weedy Rice
Source: G3 (Bethesda). 2013 Jun 1;3(6):1049–59. doi: 10.1534/g3.113.006395 (PMC3689802; doi:10.1534/g3.113.006395)
Supplement: Supporting Information [file supp_g3.113.006395_FigureS1.pdf]

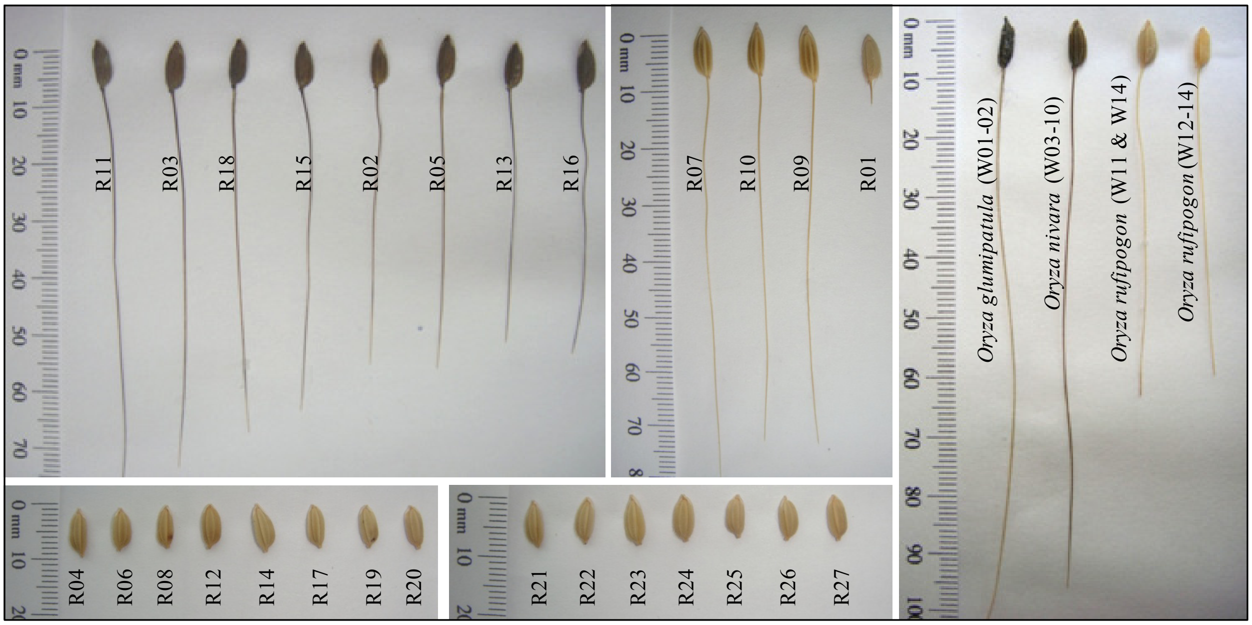

Figure S1 Seed morphologies of U.S. weedy red rice and wild rice. Refer to Table S1 for additional information about the red (R01-R27) and wild (W01-W14) rice.
