# Supplementary material for: Quantitative Trait Locus and Haplotype Analyses of Wild and Crop-Mimic Traits in U.S. Weedy Rice
Source: G3 (Bethesda). 2013 Jun 1;3(6):1049–59. doi: 10.1534/g3.113.006395 (PMC3689802; doi:10.1534/g3.113.006395)
Supplement: Supporting Information [file supp_g3.113.006395_FigureS2.pdf]

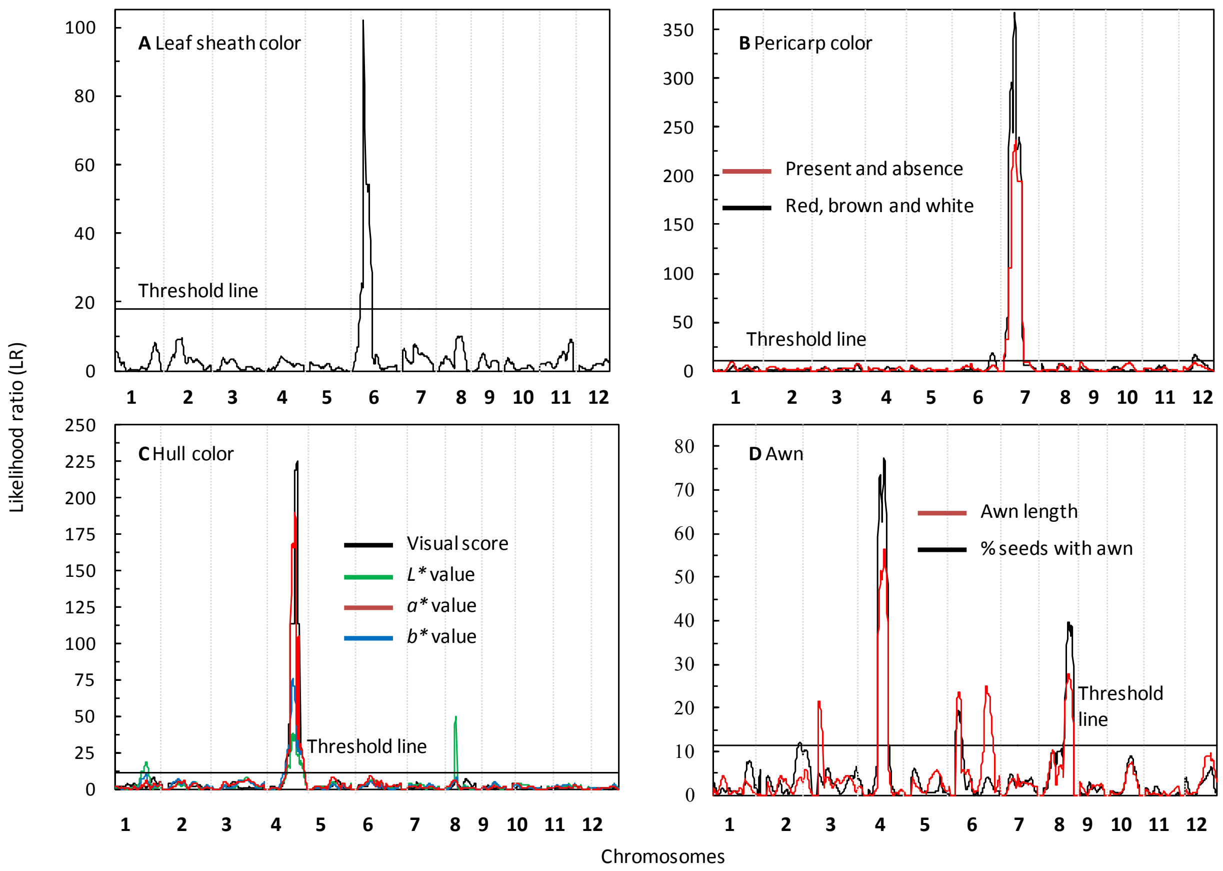

Figure S2 Genome wide scan for QTL associated with wild and crop-mimic traits in the F_2 EM93-1/US1 population. (A) Leaf sheath color (LSC); (b) pericarp color (PC), (c) hull color (HC), (d) awn (AN), (e) seed shattering (SH), (f) seed dormancy (SD), (g) flowering time (FT), (h) reproductive tiller numbers/plant (RTN), (i) plant height (PH), (j) seed weight (SW), (k) seed numbers/plant (SN) and seed setting percentage (SSP), (l) tiller number at vegetative stage (TNv) and tiller number-increasing rates (TNR) for week 6/week 4 (w6/w4) and w8/w6, (m) plant height at vegetative stage (PHv), and (n) plant height increasing rates (PHR) for for week 6/week 4 (w6/w4) and w8/w6.

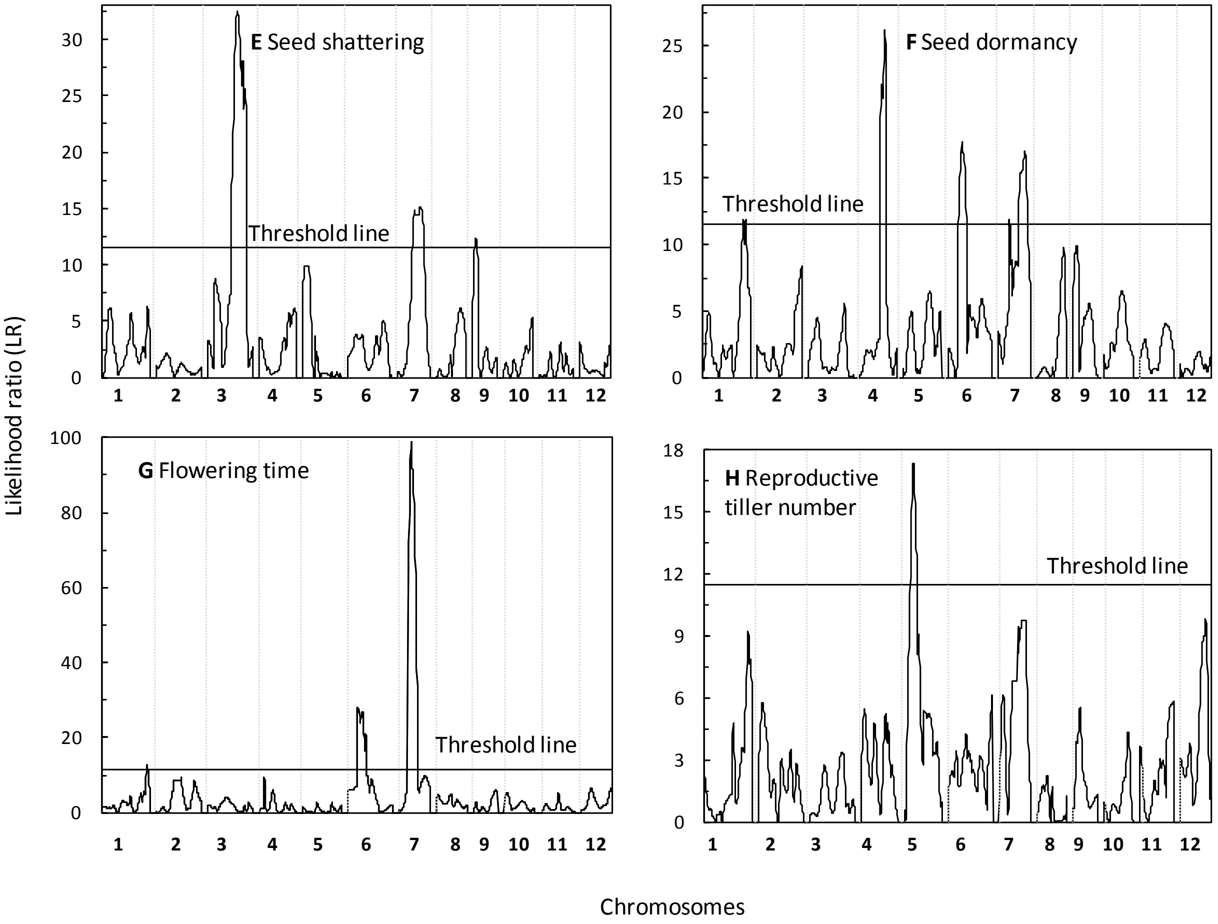

Figure S2 Continued.

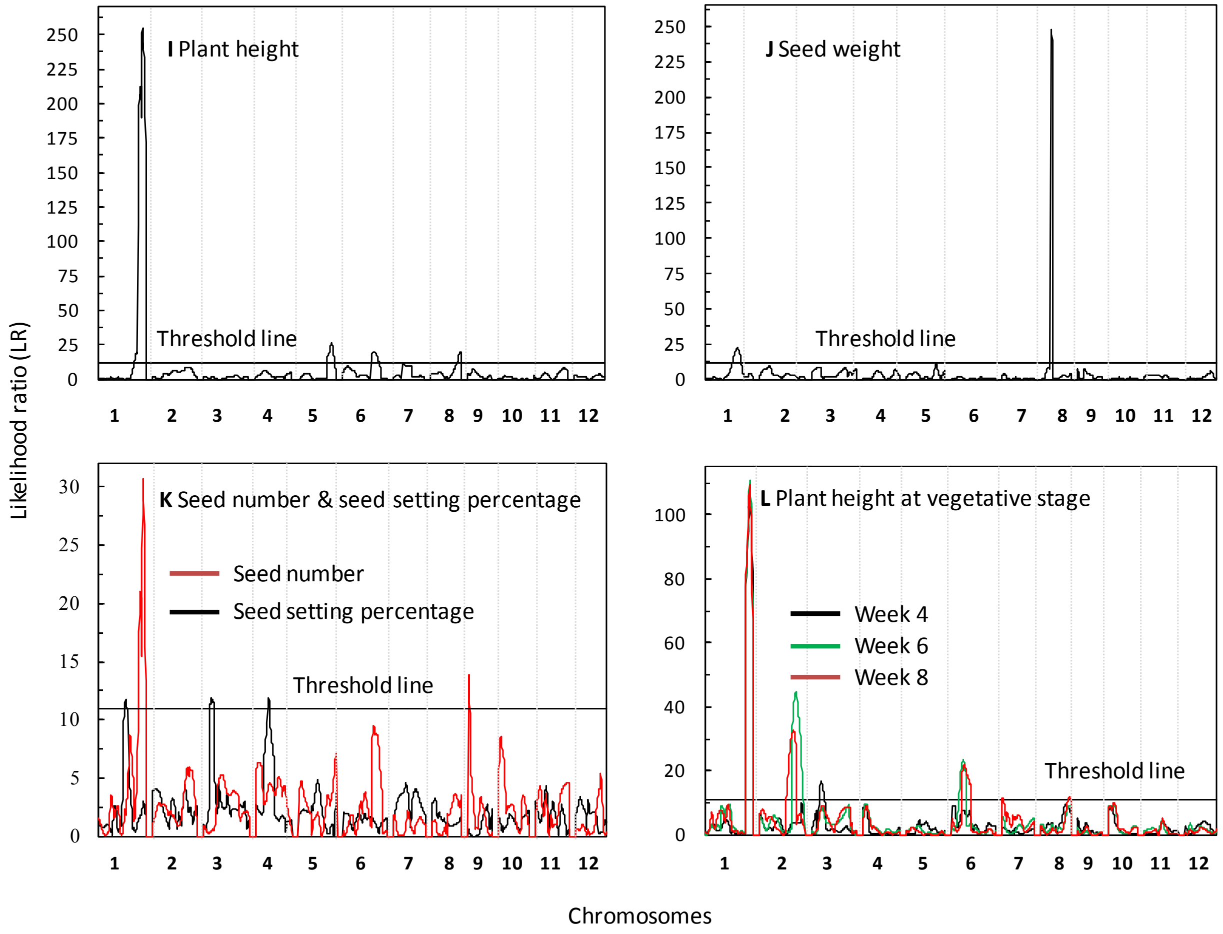

Figure S2 Continued.

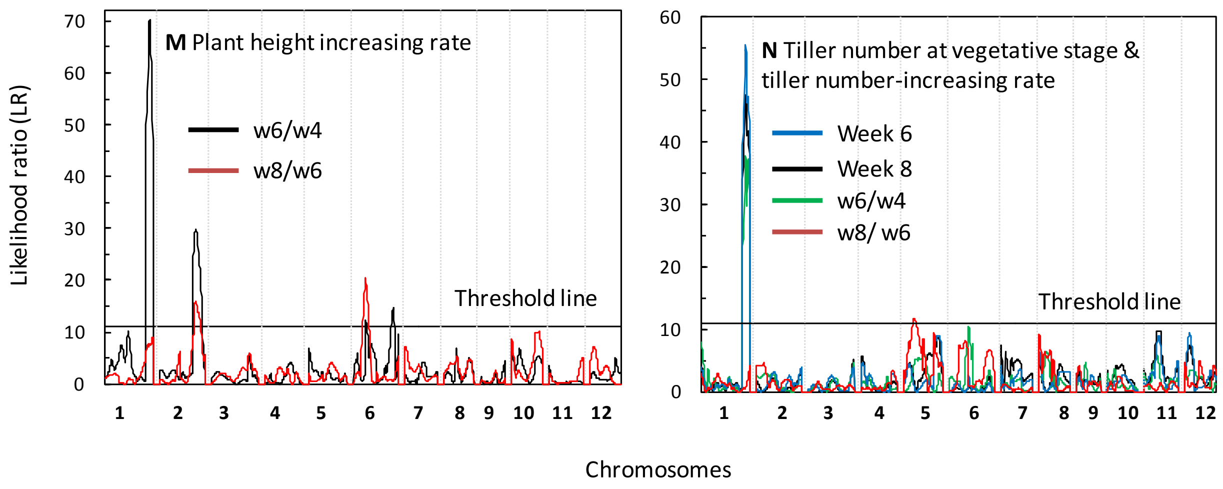

Figure S2 Continued.
