# Supplementary material for: Quantitative Trait Locus and Haplotype Analyses of Wild and Crop-Mimic Traits in U.S. Weedy Rice
Source: G3 (Bethesda). 2013 Jun 1;3(6):1049–59. doi: 10.1534/g3.113.006395 (PMC3689802; doi:10.1534/g3.113.006395)
Supplement: Supporting Information [file supp_g3.113.006395_TableS1.pdf]

Table S1 List of information on U.S. weedy red rice and wild rice lines used for this research

Code[a]	PI #[b]	Taxon[b]	Origin[b]	Phenotypes[c]				
				Hull color	Pericarp color	Awn (length in mm)	Height (cm)	Days to flowering
R01	PI-506229	*O. sativa*	California	Straw	Red	Short (<5)	66	95
R02	PI-653412	*O. sativa*	Arkansas	Black	Red	Long (35)	86	70
R03	PI-653413	*O. sativa*	Arkansas	Black	Red	Long (55)	118	97
R04	PI-653414	*O. sativa*	Arkansas	Straw	Red	None	100	86
R05	PI-653415	*O. sativa*	Arkansas	Black	Red	Long (45)	83	117
R06	PI-653416	*O. sativa*	Mississippi	Straw	Red	None	104	131
R07	PI-653417	*O. sativa*	Arkansas	Furrowed	Red	Long (60)	98	97
R08	PI-653418	*O. sativa*	Mississippi	Straw	Red	None	100	131
R09	PI-653420	*O. sativa*	Louisiana	Furrowed	Red	Long (60)	102	100
R10	PI-653421	*O. sativa*	Mississippi	Furrowed	Red	Long (55)	103	100
R11	PI-653422	*O. sativa*	Arkansas	Black	Red	Long (55)	120	105
R12	PI-653423	*O. sativa*	Arkansas	Straw	Red	None	104	86
R13	PI-653425	*O. sativa*	Arkansas	Black	Red	Long (30)	119	100
R14	PI-653426	*O. sativa*	Missouri	Straw	Red	None	99	78
R15	PI-653427	*O. sativa*	Arkansas	Black	Red	Long (40)	110	96
R16	PI-653428	*O. sativa*	Arkansas	Black	Red	Long (25)	128	96
R17	PI-653429	*O. sativa*	Arkansas	Straw	Red	None	97	80
R18	PI-653430	*O. sativa*	Arkansas	Black	Red	Long (55)	83	117
R19	PI-653431	*O. sativa*	Arkansas	Straw	Red	None	105	70
R20	PI-653432	*O. sativa*	Missouri	Straw	Red	None	105	80
R21	PI-653433	*O. sativa*	Arkansas	Straw	Red	None	90	90
R22	PI-653434	*O. sativa*	Arkansas	Straw	Red	None	97	90
R23	PI-653435	*O. sativa*	Arkansas	Straw	Red	None	90	79
R24	PI-653436	*O. sativa*	Arkansas	Straw	Red	None	94	79

R25	PI-653437	*O. sativa*	Louisiana	Straw	Red	None	62	70
R26	PI-653438	*O. sativa*	Mississippi	Straw	Red	None	72	76
R27	PI-653439	*O. sativa*	Louisiana	Straw	Red	None	75	80
Wild rice								
W01	PI-590413	*O. glumipatula*	N/A	Black	Red	Long (95)	127	N/A
W02	PI-527364	*O. glumipatula*	Brazil	Black	Red	Long (95)	132	N/A
W03	PI-590426	*O. nivara*	N/A	Black	Red	Long (85)	78	N/A
W04	PI-590404	*O. nivara*	India	Black	Red	Long (85)	90	N/A
W05	PI-590409	*O. nivara*	India	Black	Red	Long (85)	92	N/A
W06	PI-590411	*O. nivara*	India	Black	Red	Long (85)	90	N/A
W07	GSOR-311698	*O. nivara*	India	Black	Red	Long (85)	105	N/A
W08	PI-590425	*O. nivara*	Myanmar	Black	Red	Long (80)	85	N/A
W09	GSOR-311699	*O. nivara*	Myanmar	Black	Red	Long (80)	127	N/A
W10	PI-590410	*O. nivara*	N/A	Black	Red	Long (80)	132	N/A
W11	PI-590418	*O. rufipogon*	Myanmar	Furrowed	Red	Long (55)	148	N/A
W12	PI-590422	*O. rufipogon*	Myanmar	Straw	White	Long (55)	93	N/A
W13	GSOR-311703	*O. rufipogon*	Myanmar	Straw	White	Long (55)	105	N/A
W14	PI-590417	*O. rufipogon*	Taiwan	Furrowed	Red	Long (55)	142	N/A

[a] Coded for citation in this research (*e.g.,* Fig. 4).

[b] Information from the National Small Grains Collection (NSGC), USDA-ARS.

[c] Observed for plants grown in the greenhouse after the introduction from NSGC. N/A, not available.
