# Supplementary material for: Quantitative Trait Locus and Haplotype Analyses of Wild and Crop-Mimic Traits in U.S. Weedy Rice
Source: G3 (Bethesda). 2013 Jun 1;3(6):1049–59. doi: 10.1534/g3.113.006395 (PMC3689802; doi:10.1534/g3.113.006395)
Supplement: Supporting Information [file supp_g3.113.006395_TableS2.pdf]

Table S2 List of markers with a segregation ratio deviated from the expected 1:2:1 in the F_2 EM93-1/US1 population

Markers	Chr.	Position	Genotypic frequency			Fitness test (χ^2 value)*	Allelic frequency	
			EM93-1-like	Heterozygote	US1-like		EM93-1-like	US1-like
RM3740	1	22.8	0.33	0.49	0.18	8.43	0.57	0.43
RM84	1	30.9	0.32	0.51	0.18	7.78	0.57	0.43
RM283	1	41.2	0.31	0.52	0.17	7.95	0.57	0.43
RM7479	7	14.0	0.35	0.47	0.18	12.12	0.59	0.41
RM1253	7	29.4	0.29	0.54	0.16	7.49	0.56	0.44
RID12	7	34.0	0.34	0.50	0.16	12.30	0.59	0.41
RM6018	7	38.6	0.32	0.53	0.15	10.76	0.58	0.42
RM3635	7	40.2	0.31	0.52	0.16	8.68	0.57	0.43
RM3755	7	43.2	0.30	0.52	0.18	6.47	0.56	0.44
RM346	7	62.6	0.26	0.59	0.16	8.89	0.55	0.45
RM6403	7	71.1	0.31	0.53	0.15	10.34	0.58	0.42
RM38	8	10.3	0.12	0.54	0.34	18.93	0.39	0.61
RM3778	8	23.2	0.06	0.60	0.34	35.35	0.36	0.64
RM6208	8	40.2	0.01	0.48	0.51	96.20	0.25	0.75
RM3395	8	48.9	0.03	0.52	0.45	68.43	0.29	0.71
RM404	8	51.6	0.03	0.53	0.44	62.21	0.30	0.70
RM515	8	65.8	0.10	0.53	0.37	29.53	0.36	0.64
RM5515	9	8.7	0.16	0.55	0.29	8.37	0.43	0.57
RM296	9	21.5	0.17	0.55	0.28	6.41	0.44	0.56
RM6839	9	31.2	0.16	0.52	0.31	8.68	0.43	0.57
RM239	10	24.0	0.18	0.53	0.30	6.16	0.44	0.56
RM20	11	0	0.27	0.60	0.13	14.09	0.57	0.43
RM167	11	18.0	0.27	0.56	0.17	6.51	0.55	0.45
RM7283	11	37.2	0.23	0.61	0.16	11.18	0.53	0.47
RM7003	12	32.6	0.23	0.60	0.16	9.48	0.53	0.47

***** Chi-square value at *P*=0.05 is 5.99.
