# Supplementary material for: Quantitative Trait Locus and Haplotype Analyses of Wild and Crop-Mimic Traits in U.S. Weedy Rice
Source: G3 (Bethesda). 2013 Jun 1;3(6):1049–59. doi: 10.1534/g3.113.006395 (PMC3689802; doi:10.1534/g3.113.006395)
Supplement: Supporting Information [file supp_g3.113.006395_TableS3.pdf]

Table S3 Summary of observed and predicted pairs of trait correlation in the F_2 population

Cluster[a]	No. of QTL[b]	Expected QTL pairs[b]	Observed trait pairs[c]	Predicted trait pairs with[d]	
				positive corr.	negative corr.
CL1.1	3	3	1	1	2
CL1.2	8	28	16	13	15
CL2	3	3	2	1	2
CL3	3	3	2	3	0
CL4.1	2	1	1	1	0
CL4.2	2	1	1	1	0
CL5	2	1	1	1	0
CL6.1	6	15	10	7	8
CL6.2	4	6	4	2	4
CL7.1	3	3	3	3	0
CL7.2	2	1	1	1	0
CL8.1	2	1	0	1	0
CL8.2	3	3	2	3	0
CL9	2	1	1	1	0
Total[e]	45	70	45 (64%)	39 (56%)	31 (44%)

[a] Refer to Fig. 3 for map positions.

[b] Number of QTL and expected QTL pairs in the cluster.

[c] Observed pairs of trait correlation (Table 2).

[d] Predicted based on signs (+/-) of QTL additive effect (*a*) values (Tables 3-5): positive correlation, both QTLs had plus or minus *a*; negative correlation, one QTL had plus while the other had minus *a*.

[e] Percentages are based on the number of expected QTL pairs[b].
